# Increased mtDNA mutation frequency in oocytes causes epigenetic alterations and embryonic defects

**DOI:** 10.1093/nsr/nwac136

**Published:** 2022-07-13

**Authors:** Longsen Han, Yujia Chen, Ling Li, Chao Ren, Haichao Wang, Xinghan Wu, Juan Ge, Wenjie Shu, Minjian Chen, Qiang Wang

**Affiliations:** State Key Laboratory of Reproductive Medicine, Suzhou Municipal Hospital, Nanjing Medical University, Nanjing 211166, China; State Key Laboratory of Reproductive Medicine, Suzhou Municipal Hospital, Nanjing Medical University, Nanjing 211166, China; State Key Laboratory of Reproductive Medicine, Suzhou Municipal Hospital, Nanjing Medical University, Nanjing 211166, China; Department of Human Anatomy and Histoembryology, Nanjing University of Chinese Medicine, Nanjing 210033, China; Beijing Institute of Microbiology and Epidemiology, Beijing 100850, China; State Key Laboratory of Reproductive Medicine, Suzhou Municipal Hospital, Nanjing Medical University, Nanjing 211166, China; State Key Laboratory of Reproductive Medicine, Suzhou Municipal Hospital, Nanjing Medical University, Nanjing 211166, China; State Key Laboratory of Reproductive Medicine, Suzhou Municipal Hospital, Nanjing Medical University, Nanjing 211166, China; Beijing Institute of Microbiology and Epidemiology, Beijing 100850, China; State Key Laboratory of Reproductive Medicine, Suzhou Municipal Hospital, Nanjing Medical University, Nanjing 211166, China; Key Laboratory of Modern Toxicology of Ministry of Education, School of Public Health, Nanjing Medical University, Nanjing 211166, China; State Key Laboratory of Reproductive Medicine, Suzhou Municipal Hospital, Nanjing Medical University, Nanjing 211166, China; Center for Global Health, School of Public Health, Nanjing Medical University, Nanjing 211166, China

**Keywords:** mtDNA, metabolism, oocyte, embryo, epigenetics

## Abstract

Mitochondria are essential for female reproductive processes, yet the function of mitochondrial DNA (mtDNA) mutation in oocytes remains elusive. By employing an mtDNA mutator (Polg^m^) mouse model, we found the fetal growth retardation and placental dysfunction in post-implantation embryos derived from Polg^m^ oocytes. Remarkably, Polg^m^ oocytes displayed the global loss of DNA methylation; following fertilization, zygotic genome experienced insufficient demethylation, along with dysregulation of gene expression. Spindle–chromosome exchange experiment revealed that cytoplasmic factors in Polg^m^ oocytes are responsible for such a deficient epigenetic remodeling. Moreover, metabolomic profiling identified a significant reduction in the α-ketoglutarate (αKG) level in oocytes from Polg^m^ mice. Importantly, αKG supplement restored both DNA methylation state and transcriptional activity in Polg^m^ embryos, consequently preventing the developmental defects. Our findings uncover the important role of oocyte mtDNA mutation in controlling epigenetic reprogramming and gene expression during embryogenesis. αKG deserves further evaluation as a potential drug for treating mitochondrial dysfunction-related fertility decline.

## INTRODUCTION

The oocyte is the key determinant of embryo developmental competence in female reproduction. Besides delivering half the chromosomal complement to the embryo through meiosis, the oocyte also retains mitochondria, mRNAs and proteins by asymmetric division, to initiate and sustain embryo development [1]. Mitochondria are dynamic organelles essential for cell survival, death and differentiation. Although they are the main source of ATP production via oxidative phosphorylation (OXPHOS), they house a myriad other biochemical pathways among diverse cellular processes, including fatty acid oxidation, phospholipid biosynthesis and reactive oxygen generation [2,3]. Mitochondria contain their own DNA (mtDNA)—a circular molecule of ∼16 kilobases encoding 37 genes: 13 for subunits of respiratory complexes, 22 for mitochondrial tRNA and 2 for rRNA [4]. In almost all metazoans, mtDNA is exclusively maternally inherited, namely only oocyte mitochondria can be passed on to progeny [5–7]. Instead of histones, mtDNA is tightly packed with mitochondrial transcription factor A (TFAM) [8], which makes the mitochondrial genome more vulnerable to mutations caused by metabolic and environmental sources [9]. Therefore, mtDNA mutation rate is ∼9–25 times higher than that of nuclear-DNA (nDNA) [10]. On the other hand, mitochondrial genes have no introns and intergenic sequences are absent or limited to a few bases. Any mutation that occurs in mtDNA may disrupt the electron transport chain as a whole, resulting in a potential functional decline [11]. Recent studies strongly demonstrate that mtDNA–nDNA interactions profoundly affect mammalian biology [12] and contribute to some pathologies [13]. Of note, increased mtDNA mutations have been detected in germ cells exposed to maternal hyperglycemia and aging [14,15], although their consequences are undefined.

Mitochondria function as central metabolic hubs providing specific metabolites required to activate the epigenetic modifiers that regulate the deposition and removal of DNA and histone marks [16]. Progressive gain of DNA methylation is predominantly mediated by DNMT3A during oocyte growth [17]. After fertilization, Fe(II) and α-ketoglutarate (αKG)-dependent dioxygenase TET3 is responsible for the global demethylation in zygotes [18]. Thus, oocyte and early embryo development may represent sensitive windows during which mitochondria have profound effects on offspring health. However, the evidence that directly links mitochondrial function to epigenetic state in oocytes remains limited. Mice expressing a proofreading-deficient version of DNA polymerase gamma (Polg^m^ mouse), the catalytic subunit of mtDNA polymerase, accumulated 3- to 5-fold increase in spontaneous mtDNA mutation, showing reduced lifespan and declined fertility [3,19]. In the present study, by employing this mouse model, we found that mtDNA mutations in oocytes induce defective phenotypes in post-implantation embryos. Furthermore, we discovered the global loss of DNA methylation in Polg^m^ oocytes and insufficient 5mC oxidation following fertilization. Importantly, metabolomic profiling identified that metabolic dysfunction in Polg^m^ oocytes, specifically α-ketoglutarate (αKG) reduction, contributes to epigenetic alterations and developmental failure of embryos.

## RESULTS

### Premature loss of fertility in Polg^m^ mice

**Figure 1.**
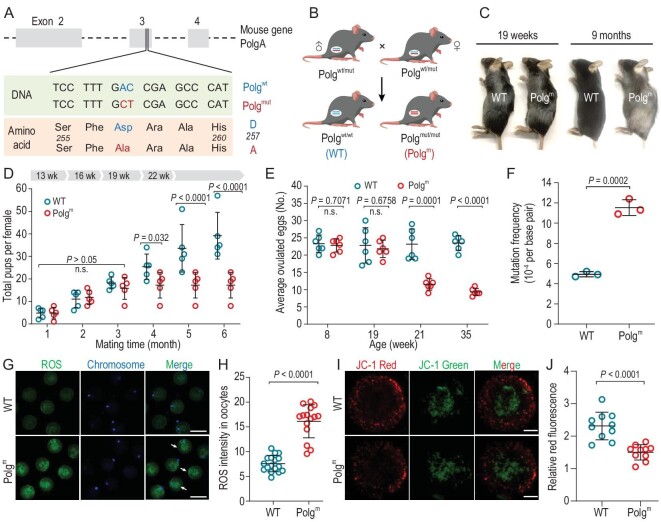
Premature loss of fertility in Polg^m^ mice. (A) Structure of *PolgA* gene and mutation strategy. Vector containing an AC→CT two-base substitution at the positions 1054–1055 in exon 3 was targeted to the mouse DNA polymerase γ (Polg). The point mutations create Asp to Ala (D→A) amino acid substitution in the conserved exonuclease domain of Polg, impairing its proofreading ability. (B) Intercrossing of mice heterozygous for the mtDNA mutator allele (Polg^wt/mut^) generates Polg^m^ (Polg^mut/mut^) and WT (Polg^wt/wt^) mice. (C) Representative images of female WT and Polg^m^ mice at 19 weeks and 9 months of age. (D) Breeding assays were performed to detect the fertility of female WT and Polg^m^ mice. Mice from WT and Polg^m^ groups were mated with stud males at 8 weeks of age, and litter and litter size were counted every month. Continuous breeding assessment showed the cumulative number of progeny per female for 6 months (five mice of each group). (E) The results of superovulation assay from WT and Polg^m^ mice at indicated age. Data are shown as mean ± SD (two mice for each experiment and six replicates). (F) The frequency of mtDNA mutation in oocytes from WT and Polg^m^ mice (20 oocytes from five mice in each experiment). (G) Representative images of ROS fluorescence (green) in WT and Polg^m^ oocytes. Scale bar, 100 μm. (H) Quantification of the relative levels of ROS in oocytes. Each data point represents an oocyte (*n* = 17 for WT, *n* = 15 for Polg^m^). (I) Representative images of mitochondrial membrane potential measured using JC-1 dye. JC-1 aggregates when the mitochondrion has a high ΔΨm (red); when mitochondrial membrane potential is disturbed, JC-1 remains in its monomeric form (green). Scale bar, 20 μm. (J) Quantification of the ratio of red:green fluorescence in WT and Polg^m^ oocytes (*n* = 10 for WT, *n* = 10 for Polg^m^). MII oocytes were obtained from 19- to 21-week-old mice for all experiments. A Student's *t-*test (two-tailed) was used for statistical analysis; n.s., not significant.

To determine whether mtDNA mutation in oocytes influences female reproduction, we used the mtDNA mutator mouse—a model that introduced an AC–CT two-base substitution at positions 1054–55 of PolgA exon 3 (Fig. 1A). This mutation strategy creates an amino acid substitution, D(aspartate)257A(alanine), in the exonuclease domain II of Polg, ablating proofreading ability without significantly affecting mtDNA replication [20]. Here, homozygous Polg^D257A/D257A^ mice (Polg^mut/mut^; abbreviated as Polg^m^ in the paper) were obtained from intercross of heterozygotes (Polg^wt/mut^) (Fig. 1B) and their oocytes/embryos are termed ‘Polg^m^ oocytes/embryos’. As early as 9 months of age, Polg^m^ mice showed accelerated aging phenotypes, such as hair loss, graying and kyphosis (Fig. 1C), consistent with previous reports [3,19]. Continuous breeding test and superovulation were performed to evaluate the female fertility. As shown in Fig. 1D, there was no significant difference in litter size between wild-type (WT) and Polg^m^ mice during the first three matings. In contrast, from 19 weeks, Polg^m^ female mice had lower litter size relative to WTs. However, we found that Polg^m^ mice experienced a dramatic drop in ovulation number from 21 weeks onwards; similar oocyte yield was observed between the two groups before this time point (Fig. 1E). These data indicate that oocyte quality from Polg^m^ mice at 19 weeks of age may be compromised, leading to the premature loss of fertility.

### Evaluation of oocyte quality from Polg^m^ mice

Next, we systematically assessed oocyte quality by isolating the ovulated eggs from 19-week-old Polg^m^ female mice. As shown in Supplementary Fig. S1A, we did not observe gross morphological differences between WT and Polg^m^ oocytes. Nonetheless, sequencing analysis showed that oocytes derived from Polg^m^ mice have an increased rate (∼2 times) of mtDNA point mutations in comparison to WT oocytes (*P* = 0.0002, Fig. 1F). On the other hand, the mtDNA copy number in Polg^m^ oocytes was comparable to WT counterparts (Supplementary Fig. S1B), implying that the exonuclease deficiency in PolgA has little effect on its DNA polymerase activity. Thus, the Polg^m^ mouse provides an excellent model to determine the roles of mtDNA mutation during oocyte/embryo development. Confocal microscopy coupled with quantitative analysis showed that both Polg^m^ and WT metaphase II (MII) oocytes display a typical barrel-shaped spindle and well-aligned chromosomes (Supplementary Fig. S1C and D). To check whether the mitochondrial dynamic was disturbed due to the increased mtDNA mutation, MitoTracker labeling was conducted on WT and Polg^m^ MII oocytes, and similar proportions of the polarized distribution pattern were observed (Supplementary Fig. S1E and F). Albeit a slight decrease, no significant difference in the bulk intracellular ATP content was detected between the two groups (Supplementary Fig. S1G). The major source of reactive oxygen species (ROS) is mitochondrial oxidative respiration [21,22]. Excessive ROS exposure could result in damage to cellular proteins, lipids and nucleic acids [23]. Hence, we decided to assess whether mtDNA mutations influence redox homeostasis in oocytes. To do this, live MII oocytes were stained with CM-H2DCFDA, which fluoresces upon oxidation by ROS. Of note, we detected a marked increase in CM-H2DCFDA fluorescence in Polg^m^ oocytes relative to WT oocytes (*P* < 0.0001, Fig. 1G)and H), indicative of the elevated ROS levels. Live-cell imaging was performed to measure mitochondrial membrane potential (ΔΨm) with JC-1 (Fig. 1I). Quantitative analysis showed that Polg^m^ oocytes had a lower red:green fluorescence ratio (*P* < 0.0001, Fig. 1J), indicating the reduced ΔΨm. Collectively, these data suggest that overload of mtDNA mutation significantly impairs mitochondrial function in oocytes.

### Developmental defects of post-implantation embryos derived from Polg^m^ oocytes

**Figure 2.**
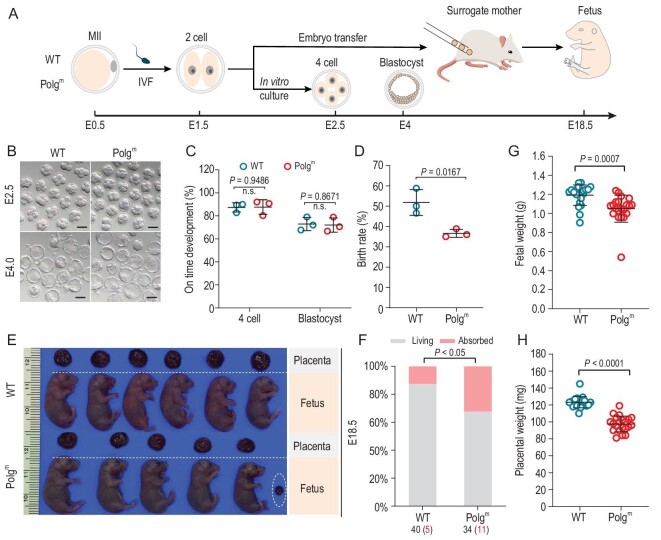
Developmental defects of post-implantation embryos derived from Polg^m^ oocytes. (A) Schematic diagram of *in vitro* fertilization (IVF) and embryo transfer experiments. Embryonic days corresponding to the developmental stages of embryos are indicated. (B) Representative bright-field images of Polg^m^ oocyte-derived E2.5 and E4 embryos. Scale bar, 50 μm. Oocytes (*n* = 35 for WT, *n* = 42 for Polg^m^) used for IVF were collected from three mice. (C) The percentage of Polg^m^ oocyte-derived embryos that successfully progressed to the four-cell and blastocyst stage at E2.5 and E4 during *in vitro* culture. (D) The birth rate of Polg^m^ oocyte-derived two-cell embryos that were transferred to surrogate mothers (three surrogate ICR mothers were used for each group and 15 embryos per surrogate mouse). (E) Representative images of Polg^m^ oocyte-derived fetuses and placentas at E18.5 by performing caesarian section. Dash circle indicates the absorbed embryo. (F) Quantification of living and absorbed embryos at E18.5. Implantation sites without visible embryos were regarded as ‘absorbed’. (G) and (H) Quantification of E18.5 fetal and placental weight derived from WT and Polg^m^ oocytes. Three or four surrogate mothers were used for fetal/placental evaluation. Nineteen- to 21-week-old mice were used for superovulation and IVF. Data are expressed as mean ± SD. Student's *t-*test (two-tailed) was used for statistical analysis; n.s., not significant.

The above findings prompted us to consider whether the impaired oocyte quality results in fertility loss of the Polg^m^ mouse by disrupting embryogenesis. To exclude the possible effects of the Polg^m^ uteri environment on embryonic development, we carried out *in vitro* fertilization (IVF) of oocytes derived from WT and Polg^m^ mice, and then examined the phenotypes of pre-implantation embryos during *in vitro* culture and post-implantation embryos following embryo transfer to surrogate mothers (Fig. 2A). Early embryos from Polg^m^ oocytes exhibited similar on-time progression to four-cell and blastocyst stages relative to WTs at E2.5 and E4 during *in vitro* culture, as described previously [24] (Fig. 2B)and C). However, when these two-cell embryos were transferred into the oviducts of pseudo-pregnant females, Polg^m^ oocyte-derived embryos yielded the significantly lower live birth rate than WT embryos (*P* = 0.0167, Fig. 2D). Moreover, we evaluated fetal growth *in uteri* by performing caesarian section at E18.5. As shown in Fig. 2E)and F, developmental failure was observed in Polg^m^ embryos, as evidenced by the increased frequency of absorbed fetuses. Remarkably, the weight of fetuses originated from Polg^m^ oocytes was diminished by ∼15%–20% (*P* = 0.0007, Fig. 2E)and G), indicating fetal growth retardation. In addition, we noticed that placentas derived from Polg^m^ embryos also displayed weight reduction (∼15%–20%) compared to their WT counterparts (*P* < 0.0001, Fig. 2E)and H, circle). Altogether, the results suggest that mtDNA mutations in oocytes lead to developmental defects of post-implantation embryos and placental dysfunction, consequently compromising female fertility.

### Global loss of DNA methylation in Polg^m^ oocytes

**Figure 3.**
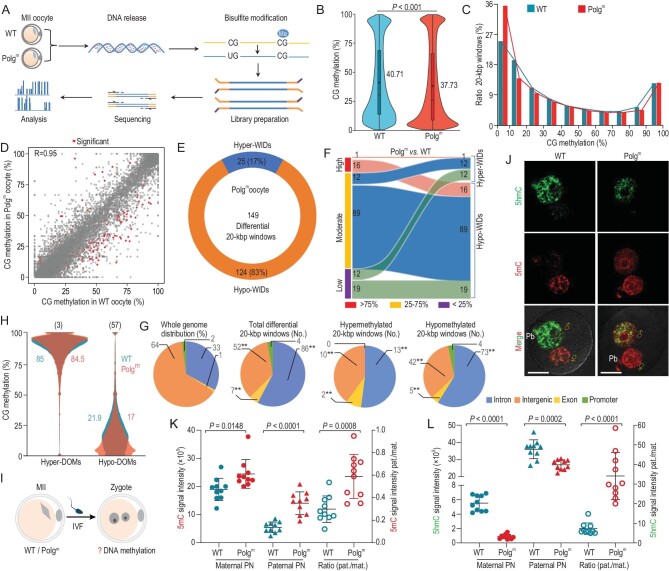
Altered DNA methylation in oocytes and zygotes derived from Polg^m^ mice. (A) Flow chart of whole-genome bisulfite sequencing. MII oocytes without cumulus cells contamination were collected from 19- to 21-week-old mice and prepared for methylome analysis (two biological replicates for each group, *n* = 20 oocytes for each replicate). (B) Violin plots showing the methylation levels of WT and Polg^m^ oocytes. Mean methylation levels are marked by the numerical value and black cross. Boxes represents the interquartile range. Statistical analysis was performed with a bootstrap test to detect the significance of CG methylation distribution between the two groups. (C) Distribution of 20-kbp genomic windows in Polg^m^ and WT oocytes according to their percentage of DNA methylation. (D) Scatter plot of average DNA methylation levels for 20-kbp windows between WT and Polg^m^ oocytes. Differentially methylated 20-kbp windows are shown in red dots. The *R*-value is denoted in the top left corner. (E) Circular representation of the proportion of hyper- and hypomethylated 20-kbp windows (Hyper-WIDs and Hypo-WIDs) between WT and Polg^m^ oocytes. (F) River plot illustrating the distribution of CpG methylation in Hyper-WIDs and Hypo-WIDs. High: >75%; moderate: 25–75%; low: <25%. (G) Pie charts illustrating the relative proportion of differential 20-kbp windows among different genomic elements. A Fisher's exact test was performed to evaluate the potential enrichment or depletion of differential windows relative to the genomic distribution of the elements: **P < 0.01. (H) Violin plots of the distribution of average CG methylation values across hypermethylated domains (Hyper-DOMs) and hypomethylated domains (Hypo-DOMs) in WT and Polg^m^ oocytes. Mean methylation levels are indicated by the numerical values. The number of differentially methylated domains are denoted in the top. (I) Schematic diagram of epigenetic analysis in zygotes. Nineteen- to 21-week-old mice were used for superovulation and IVF. (J) Late-stage zygotes from both groups were stained with anti-5mC (red) and anti-5hmC (green) antibodies. ♂ and ♀ indicate the paternal PN and maternal PN, respectively. Pb, polar body. Scale bars, 25 μm. (K) and (L) Quantification of the levels of fluorescence signals for 5mC and 5hmC in zygote pronuclei. Results are presented as integrated signal intensity in the paternal (pat.) and maternal (mat.) PN (left axis) or a ratio of the signal for the paternal and maternal PN (right axis). Each point represents one zygote. *n* = 10 for each group. Data are expressed as mean ± SD. Student's *t-*test (two-tailed) was used for statistical analysis; n.s., not significant.

Improper epigenetic modifications in oocytes and embryos have been implicated as the major cause of post-implantation developmental defects [25–27]. Given the findings mentioned above, we therefore asked whether the DNA methylation establishment in Polg^m^ oocytes was disturbed. To address this question, using whole-genome bisulfite sequencing (BS-Seq) method for small samples [28], base-resolution methylomes of MII oocytes were obtained (Fig. 3A). Of note, Polg^m^ oocytes showed a significant decrease in global CpG methylation levels relative to WT oocytes (*P* < 0.001, 37.7% Polg^m^ vs. 40.7% WT; Fig. 3B). This hypomethylation pattern was detected across the majority of genomic features examined, such as promoter, intron, untranslated region (UTR) and, particularly, the repetitive elements including transposon, long interspersed nuclear elements (LINEs) and short interspersed nuclear elements (Supplementary Fig. S2).

To get an initial overview of the DNA methylation landscape, the distribution of average methylation levels for non-overlapping 20-kbp windows tiled through the genome was first evaluated. Consistent with previous reports [29], a bimodal pattern of CpG methylation was observed for both WT and Polg^m^ oocytes (Fig. 3C). To further determine whether certain genomic regions in Polg^m^ oocytes exhibited preferential loss of methylation, we analysed the methylome data based on several published oocyte-relevant annotations [29,30], as follows. One hundred and forty-nine differentially methylated 20-kbp windows were identified between two groups, of which 124 were hypomethylated (Hypo-WID; 83%) and 25 were hypermethylated (Hyper-WID; 17%) in Polg^m^ oocytes (Fig. 3D and E, and Supplementary Table 2). It is worth noting that most Hypo-WIDs localized in the regions with moderate methylation levels (25%–75%) (Fig. 3F). Moreover, both Hyper-WIDs and Hypo-WIDs were enriched in introns/exons and depleted from intergenic regions (Fig. 3G), indicating that they are not distributed randomly throughout the genome of Polg^m^ oocytes. On the other hand, methylated and unmethylated CpGs in oocytes tend to cluster together, establishing large-scale hypermethylated domains (Hyper-DOMs) and hypomethylated domains (Hypo-DOMs) [29]. We noticed that the mean methylation level of Hypo-DOMs (0%–25%) was substantially reduced in Polg^m^ oocytes (17.0% Polg^m^ vs. 21.9% WT; Fig. 3H). In line with this observation, 57 out of 60 (95%) differentially methylated domains were Hypo-DOMs (Supplementary Table 3). These results suggest that Polg^m^ oocytes appear to primarily lose DNA methylation in regions with low-to-moderate CG methylation levels.

Next, to dissect the altered epigenetic landscape more specifically, a search for differentially methylated regions (DMRs) between WT and Polg^m^ oocytes was conducted. We discovered a total of 87 DMRs (Supplementary Table 4), of which 35 were hypermethylated (hyper-DMRs; 40%) and 52 were hypomethylated (hypo-DMRs; 60%) in Polg^m^ oocytes (Supplementary Fig. S3A and B). To understand the potential functional involvement of methylation changes in Polg^m^ oocytes, the DMRs-associated genes were subjected to Gene Ontology (GO) analysis. Numerous genes were significantly enriched in terms like cell-cycle transition and adhesion (Supplementary Fig. S3C). Disturbance of these biological processes might influence embryo organogenesis and growth [31]. By bisulfite pyrosequencing analysis, we confirmed that two selected genome loci (*LINE-1* and *Wnt4*) involved in embryo development had significantly less methylation in Polg^m^ oocytes, consistent with the methylome data (Supplementary Fig. S3D and E). Cumulatively, the findings indicate that mtDNA mutations disrupt the methylation landscape in oocytes, which may be associated with the fetal developmental defects and placental dysfunction observed in Polg^m^ mice.

### Disrupted epigenetic reprogramming during Polg^m^ zygote development

Upon fertilization, drastic epigenetic reprogramming takes place [32]. The major initial event is global DNA demethylation in the zygote. Given the aberrant DNA methylation establishment in Polg^m^ oocytes, we further asked whether epigenetic reprogramming was also influenced during zygotic development (Fig. 3I). To address this issue, zygotes derived from WT and Polg^m^ oocytes were labeled with antibodies specific for 5mC and 5hmC [33], and then examined using confocal microscopy. In WT zygotes, we observed a rapid loss of 5mC staining and increase in 5hmC staining in paternal pronucleus (PN), whereas maternal PN constantly retained a strong 5mC signal (Fig. 3J–L). In striking contrast, the 5mC level was significantly elevated in both pronuclei of Polg^m^ zygotes (Fig. 3J–L), indicating the insufficient oxidation of 5mC to 5hmC. These observations suggest that mtDNA mutations in oocytes also affect the epigenetic reprogramming during zygotic development.

### Dysregulation of gene expression in Polg^m^ early embryos

**Figure 4.**
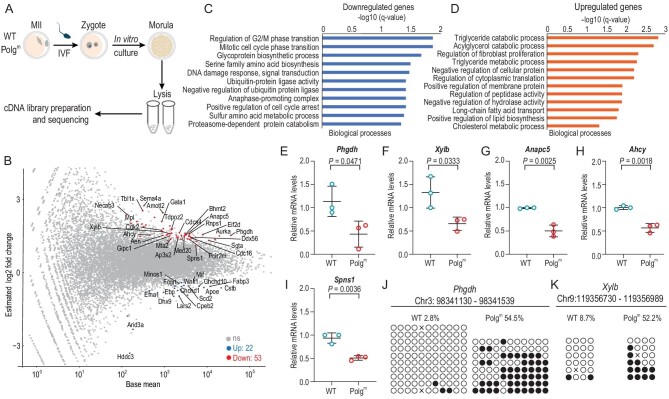
Dysregulation of gene expression in Polg^m^ embryos. (A) Schematic illustration of the experimental design. MII oocytes were collected from WT and Polg^m^ female mice at 19–21 weeks of age (seven embryos per sample, two samples for each group). (B) MA plot of gene expression changes in WT and Polg^m^ morula embryos. Significantly changed genes were selected when the adjusted *q*-value was <0.05 (blue for the upregulated genes and red for the downregulated genes). (C) and (D) Functional annotation for the downregulated and upregulated genes in Polg^m^ oocytes compared to WT cells. Significance is indicated as -log10 *q*-value. (E)–(I) Relative mRNA levels of the indicated genes in morula embryos derived from WT and Polg^m^ mice, validated by qRT-PCR. Data are presented as mean ± SD. Student's *t*-test was used for statistical analysis. (J) and (K) BS-Seq analysis examining the methylation status of *Phgdh* and *Xylb* promoters in WT and Polg^m^ morula. Circles represent CpG dinucleotides either unmethylated (open) or methylated (closed). The gene promoter positions selected for validation are indicated above.

DNA methylation is one of the main epigenetic mechanisms that regulates gene expression. To investigate whether the epigenetic abnormalities during Polg^m^ zygotic reprogramming influence the transcriptional activity in the subsequent embryo development, morula embryos derived from IVF were obtained for RNA-sequencing (RNA-Seq) (Fig. 4A). Seventy-five differentially expressed genes (DEGs) were identified in Polg^m^ embryos as compared with WT embryos, including 53 downregulated and 22 upregulated genes (Fig. 4B and Supplementary Table 5). Intriguingly, GO analysis showed that the downregulated genes in Polg^m^ embryos are primarily involved in cell-cycle regulation (Fig. 4C). By contrast, the majority of upregulated genes are enriched in metabolic process (Fig. 4D). To validate RNA-Seq data, the expression of 5 DEGs (*Phgdh, Xylb, Anapc5, Ahcy, Spns1*) associated with embryonic development [34–37] was examined by performing qRT-PCR (Fig. 4E–I). These genes were selected based on their functions in embryogenesis and metabolism [34,35,37]. For instance, AHCY, the only enzyme that catalyses the reversible hydrolysis of S-adenosylhomocysteine (SAH) to adenosine and L-homocysteine, is required for post-implantation embryo development [36]. Furthermore, bisulfite pyrosequencing assay was conducted to check whether the DNA methylation state of these genes was altered in Polg^m^ embryos correspondingly. Of note, significant gain of methylation was detected in four out of five promoter regions in Polg^m^ morula samples (Fig. 4J and K, and Supplementary Fig. S4). DNA methylation of promoters has been implicated to negatively correlate with gene expression during early embryonic development [38]. Thus, these data indicate that disruption of epigenetic reprogramming in Polg^m^ zygotes may dysregulate gene expression during embryogenesis via altering methylation levels. Nonetheless, the results presented in this study cannot rule out the possibility that other unknown mechanisms also contribute to this process.

### Deficient cytosol impairs epigenetic reprogramming in zygotes from Polg^m^ mice

**Figure 5.**
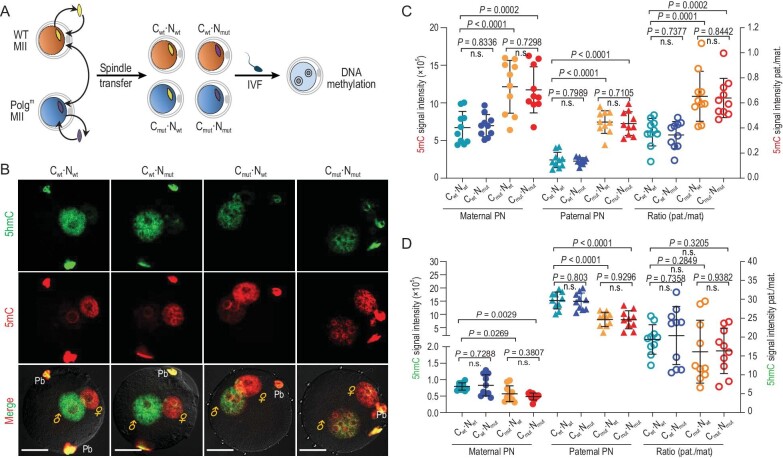
Deficient cytosol impairs epigenetic reprogramming in zygotes from Polg^m^ mice. (A) Schematic illustration of the spindle transfer experiments. Nineteen- to 21-week-old mice were used for MII oocyte collection. For reconstructed oocytes, cytoplasm derived from WT or Polg^m^ oocytes is denoted as ‘C_wt_’ or ‘C_mut_’, respectively; chromosomes derived from WT or Polg^m^ oocytes are denoted by ‘N_wt_’ or ‘N_mut_’, respectively. (B) Representative images of zygotes stained with anti-5mC (red) and anti-5hmC (green) antibodies. (C) and (D) Quantification of the levels of fluorescence signals for 5mC and 5hmC in zygotes pronuclei. Results are presented as integrated signal intensity in the paternal (pat.) and maternal (mat.) PN (left axis) or a ratio of the signal for the paternal and maternal PN (right axis). Each point represents one zygote. *n* = 10 for each group. ♂ and ♀ indicate the paternal PN and maternal PN, respectively. Pb, polar body. Scale bars, 25 μm. Data are expressed as mean ± SD. One-way ANOVA with multiple comparison was used for statistical analysis; n.s., not significant.

Next, we asked whether the abnormal epigenetic remodeling in Polg^m^ embryos is caused by faulty oocyte cytoplasm or genome, or perhaps both. To address this question, we swapped the spindle–chromosome complex between WT and Polg^m^ oocytes, followed by *in vitro* fertilization (Fig. 5A). For zygotes derived from reconstructed oocytes composed of WT cytoplasm (C) and Polg^m^ or WT nucleus (N) (termed C_wt_·N_mut_ or C_wt_·N_wt_, respectively), 5hmC staining is enriched in the paternal pronucleus (PN), whereas maternal pronucleus displays strong 5mC staining (Fig. 5B–D). Remarkably, in those zygotes derived from reconstructed oocytes composed of Polg^m^ cytoplasm and Polg^m^ or WT nucleus (termed C_mut_·N_mut_ or C_mut_·N_wt_, respectively), we found that, in both pronuclei, the 5mC signal was significantly elevated and the 5hmC level was diminished accordingly (Fig. 5B–D). Therefore, we conclude that cytosol defects, not nucleus factors, in Polg^m^ oocytes, perhaps dysfunctional mitochondria, play a major role in the aberrant epigenetic remodeling during zygotic development.

### Altered metabolite profile in Polg^m^ oocytes

**Figure 6.**
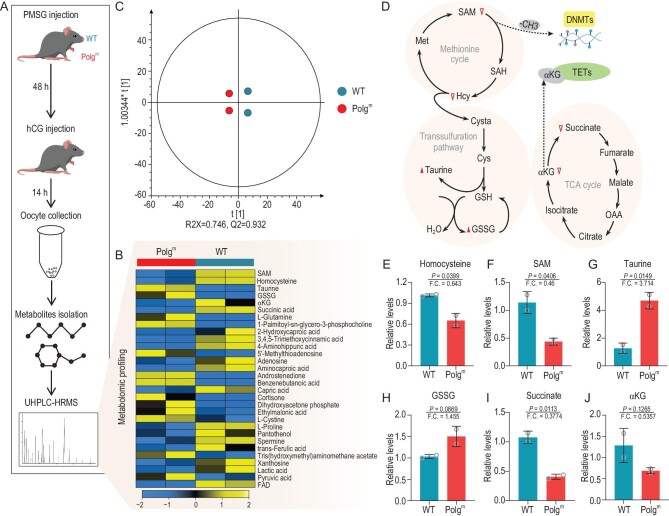
Metabolomic profiling of MII oocytes from WT and Polg^m^ mice. (A) Flow diagram of metabolome analysis in oocytes. Nineteen- to 21-week-old mice were used for MII oocyte collection (300 oocytes per sample, two samples for each group). (B) Heat map showing the 30 differential metabolites between WT and Polg^m^ oocytes. (C) OPLS-DA score plot model separating WT and Polg^m^ oocytes. R2X = 0.746, indicating 74.6% of the variation in the data set could be modeled by the selected components; Q2 = 0.932, indicating a high predictive accuracy. (D) Schematic representation of interconnected metabolic pathways associated with differentially enriched metabolites in WT and Polg^m^ oocytes. Metabolites increased in Polg^m^ oocytes are indicated by red filled triangles and metabolites decreased in Polg^m^ oocytes are marked by red empty triangles. (E)–(J) Relative levels of metabolites related to the transsulfuration pathway and TCA cycle. Student's *t*-test was used for statistical analysis in all panels.

Mitochondria are one of most characterized metabolic hubs of the cell. They produce several metabolites that serve as cofactors or substrates for epigenome modifying enzymes [16]. Epigenetic alterations in Polg^m^ oocytes/zygotes prompted us to propose that metabolic changes might be involved in this process. To test this hypothesis, we analysed the metabolite profile changes between WT and Polg^m^ oocytes using ultra-high-performance liquid chromatography-tandem high-resolution mass spectrometry (UHPLC-HRMS) (Fig. 6A). A total of 30 differential metabolites were identified from a total of 143 detectable metabolites between the WT and Polg^m^ groups (Fig. 6B), based on a *t*-test coupled with a variable importance in projection analysis (Supplementary Table 6). Robust orthogonal partial least squares-discriminant analysis (OPLS-DA) clearly separated the two groups (Fig. 6C). The homocysteine (Hcy)–methionine cycle produces universal methyl group donor S-adenosylmethionine (SAM) for a variety of reactions such as the methylation of proteins and nucleic acids, allowing modulation of their biological functions [39]. Strikingly, our metabolomic data showed that the levels of SAM and Hcy in Polg^m^ oocytes were significantly lower than that in WT oocytes (Fig. 6D–F), indicative of the downregulated methionine cycle in Polg^m^ oocytes. The transsulfuration pathway involves the interconversion of Hcy and cysteine (Cys), through the intermediate cystathionine (Cysta). Cys is oxidatively catabolized by cysteinesulfinate-dependent pathways to yield taurine and sulfate [40]. In support of this notion, we observed an increase in taurine (Fig. 6D)and G), a known product of Cys breakdown, and oxidized glutathione (GSSG) (Fig. 6D)and H), a precursor of Cys, in Polg^m^ oocytes. The transsulfuration pathway connects glutathione (GSH) synthesis to defend against oxidative stress. Taking into account the increase in the GSSG level (Fig. 6D)and H) and ROS content (Fig. 1G), the results indicate the imbalance of redox homeostasis in Polg^m^ oocytes. Moreover, we examined the expression levels of DNA methyltransferase (*Dnmt1, Dnmt3a* and *Dnmt3b*) and demethylase (*Tet3*) in WT and Polg^m^ oocytes and no significant changes were detected (Supplementary Fig. S5). In addition, one-carbon metabolism also participates in fueling methyltransferase reactions that shape the epigenetic landscape. Nevertheless, we did not see the differential mRNA expression of the relevant enzymes in this pathway (Supplementary Fig. S6). The findings above suggest that global loss of DNA methylation in Polg^m^ oocytes is probably due to the diminished methionine cycle activity and insufficient provision of methyl donors.

During mouse zygotic reprogramming, maternal and paternal genomes both undergo active demethylation by TET3 mediated 5mC to 5hmC oxidation [41]. TET3, belonging to the 2-oxoglutarate-dependent dioxygenases (2-OGDO) family, requires αKG for hydroxylation of 5mC [16]. It is worth noting that the abundance of two key components of the tricarboxylic acid (TCA) cycle (α-ketoglutarate, αKG; succinate) showed a dramatic decrease in Polg^m^ oocytes (Fig. 6D, I and J), which may contribute to the insufficient 5mC oxidation in Polg^m^ zygotes. No significant changes in the mRNA level of TCA-cycle-related enzymes were detected between WT and Polg^m^ oocytes (Supplementary Fig. S7), implying that other unknown factors probably mediate this metabolic disorder. Collectively, the findings above strongly suggest that mtDNA mutation-induced mitochondrial dysfunction, specifically the downregulated methionine cycle and TCA cycle, is likely associated with the inappropriate epigenetic modifications in oocytes and early embryos.

### **α**KG supplementation alleviates the developmental defects of Polg^m^ embryos

**Figure 7.**
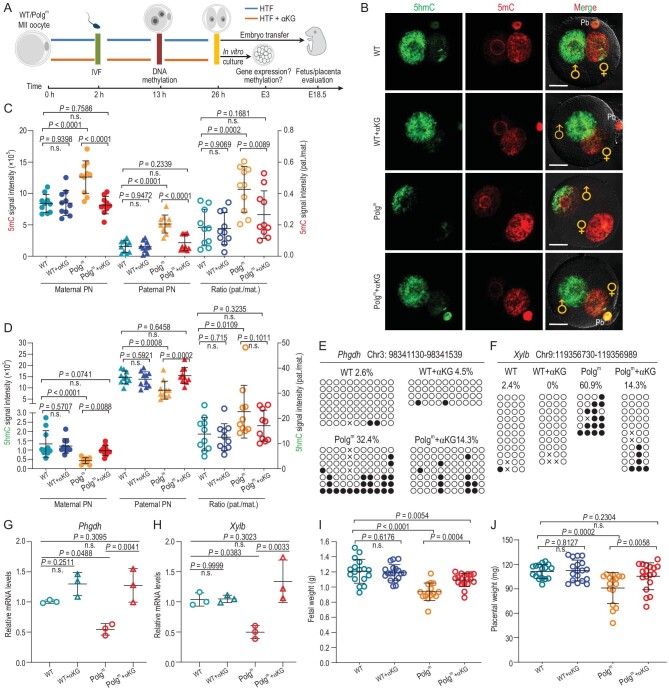
αKG supplementation alleviates the developmental defects in Polg^m^ embryos. (A) Schematic illustration of the αKG supplementation experiments. MII oocytes obtained from 19- to 21-week-old mice were cultured in HTF media supplemented with or without αKG for 2 hours before fertilization. Two-cell embryos were selected for *in vitro* culture or embryo transfer for further analysis as indicated. (B) Representative images of zygotes stained for 5mC (red) and 5hmC (green). ♂ and ♀ indicate the paternal PN and maternal PN, respectively. Pb, polar body. Scale bars, 25 μm. (C) and (D) Quantitative analysis of the fluorescence intensity of 5mC and 5hmC in zygote pronuclei. Results are presented as integrated signal intensity in the paternal (pat.) and maternal (mat.) PN (left axis) or a ratio of the signal for the paternal and maternal PN (right axis). Each point represents one zygote. *n* = 10 for each group. (E) and (F) BS-Seq analysis examining the methylation status of *Phgdh* and *Xylb* promoters in WT and Polg^m^ morula treated with or without αKG. Circles represent CpG dinucleotides either unmethylated (open) or methylated (closed). The gene promoter positions selected for validation are indicated above. (G) and (H) qRT-PCR analysis verifying the expression of *Phgdh* and *Xylb* in WT and Polg^m^ morula with or without αKG supplementation. (I) and (J) Quantification of E18.5 fetal and placental weight derived from WT and Polg^m^ embryos with or without αKG treatment. Three surrogate mothers were used for fetal/placental evaluation. Data are expressed as mean ± SD. One-way ANOVA with multiple comparisons was used for statistical analysis; n.s., not significant.

Finally, we determined whether the epigenetic/developmental abnormalities we observed in Polg^m^ embryos are due to αKG depletion in cytoplasm. For this purpose, WT and Polg^m^ oocytes at MII stage were collected and cultured in medium containing αKG and then the resultant embryos were examined as detailed in Fig. 7A. We found that αKG supplementation had little effect on WT zygotes, but significantly promoted 5mC oxidation to 5hmC in parental pronuclei of Polg^m^ zygotes (Fig. 7B–D), back to the normal pattern. This observation corroborates the idea that αKG depletion in Polg^m^ oocytes is a critical factor that leads to epigenetic alterations following fertilization. Moreover, to explore the effects of αKG supplementation on gene expression and methylation status in Polg^m^ embryos in more detail, we assayed the methylation levels and mRNA abundance of two representative genes (*Phgdh* and *Xylb*). By BS-Seq analysis, we confirmed that the selected promoter regions had significantly more methylation in Polg^m^ morula, consistent with our previous observation (Fig. 7E)and F). Although the effects of αKG supplementation on the methylation state of these regions varied, the methylation levels of both genes in Polg^m^ embryos were almost restored back to normal (Fig. 7E)and F) and the abundance of corresponding transcripts was thus markedly elevated (Fig. 7G)and H). Furthermore, the embryos were transferred into the uteri of surrogate mothers and allowed to develop until E18.5. As mentioned above (Fig. 2G)and H), fetuses and placentas originating from Polg^m^ oocytes exhibited growth retardation and weight reduction, respectively, compared with their WT counterparts. Importantly, αKG administration was able to partially prevent these fetal/placental developmental defects (Fig. 7I)and J). αKG supplementation seemed to have no significant effect on the post-implantation development of embryos derived from WT oocytes (Fig. 7I)and J). Altogether, the results demonstrate that mtDNA mutation-induced epigenetic changes and developmental failure of embryos are, at least in part, due to αKG reduction in Polg^m^ oocytes.

## DISCUSSION

The frequency of mtDNA mutation in Polg^m^ mice was ∼3–8 times higher than that in WT mice for most somatic tissues examined [3]. Here we detected an ∼2-fold increase in mtDNA mutation in Polg^m^ oocytes compared to WT oocytes. This is probably due to the purifying selection mechanism that has been reported to exist during oogenesis, restricting the transmission of deleterious mitochondria [42,43]. Besides, the WT mice used in the current study were obtained by crossing heterozygous mutant parents. The mtDNA transmitted to WT mice came from a heterozygous mutator environment, which may influence the mutation evaluation in germ cells. Interestingly, we did not see significant differences in the mtDNA copy number, mitochondrial distribution and meiotic apparatus between WT and Polg^m^ oocytes. Nonetheless, consistent with the previous report [44], we detected excessive ROS production in oocytes collected from Polg^m^ mice (Fig. 1). Glutathione is a major antioxidant that neutralizes free radicals to form water and itself then transforms to oxidative state (GSSG). Our metabolomics data identified a significant increase in the GSSG level in Polg^m^ oocytes (Fig. 6). Hcy lies at the intersection of two metabolic pathways whereby it may either be remethylated to methionine or utilized by the transsulfuration pathway to form cysteine [45]. In the present study, we found that the abundance of Hcy was decreased in Polg^m^ oocytes (Fig. 6). Although cysteine was undetectable in the metabolome profiling, taurine, a known product of cysteine breakdown, was markedly increased in mtDNA mutant oocytes. Combined with the lowered SAM level, therefore, these observations indicate that increased mtDNA mutation frequency downregulates the activity of the methionine cycle coupled with the activation of the transsulfuration pathway in mouse oocytes.

Mammalian oocytes are known to contain ∼10^5^ mtDNAs, at least an order of magnitude greater than that reported in most somatic cells [46]. It has been reported that oocytes with as few as 4000 copies of mtDNA can be fertilized and then progress normally to the blastocyst stage [46]. Therefore, the high frequency of mtDNA mutation in oocytes may be unable to induce the developmental block of pre-implantation embryos. In line with this concept, early embryos originating from Polg^m^ oocytes exhibited similar on-time progression to the blastocyst stage as compared to WTs. In contrast, fetal growth retardation and placental dysfunction were readily observed in post-implantation embryos derived from Polg^m^ oocytes (Fig. 2). Progressive gain of DNA methylation occurred during oocyte growth after birth, which is predominantly mediated by *de novo* DNA methyltransferase [47]. One of the metabolites in the methionine cycle, SAM, as the universal methyl donor, is the substrate for a host of DNA/histone methyltransferases that regulate gene expression and epigenetic inheritance [48]. Notably, genome-wide profiles reveal the global hypomethylation in Polg^m^ oocytes. The genomic regions with low-to-moderate methylation levels displayed preferential loss of methylation (Fig. 3). Mutations in the mtDNA have been found to induce distinct metabolic changes at different heteroplasmy levels, causing transcriptional variability [49]. On the other hand, transcription is a cornerstone of DNA methylation establishment in female germ cells [29]. Reduced methylation in these regions may lead to detrimental chromosomal rearrangements and misregulated gene activity [50]. For example, retrotransposon upregulation is associated with placental abnormalities in the junctional zone and labyrinth, contributing to reduced placental weight and intrauterine growth defects [51], similar to the phenotypes we observed in Polg^m^ mice.

On the other hand, mitochondria produce numerous metabolites from the TCA cycle, fatty acid oxidation and ketogenesis. They have been identified as cofactors or substrates for epigenetic modifying enzymes [52]. For instance, Bao *et al*. demonstrated that mitochondrial respiratory chain dysfunction induced by mtDNA depletion leads to alternations in one-carbon metabolism pathways [53]. Mitochondrial dysfunction induced by mtDNA depletion also results in a cell-wide metabolic rewiring primarily centered on amino acids, which altered nuclear-DNA methylation by affecting methionine and polyamine metabolism [54]. αKG, an endogenous intermediary metabolite in the TCA cycle, is required for TET-mediated DNA demethylation [55]. Insufficient 5mC oxidation caused by TET3 deletion during zygotic reprogramming has been demonstrated to be able to impair post-implantation embryo development [56]. Here, we first observed a significant increase in 5mC staining in both pronuclei of zygotes derived from Polg^m^ oocytes (Fig. 3). Of note, this incomplete demethylation is likely associated with the dysregulation of gene expression in Polg^m^ embryos, evidenced by RNA-Seq analysis (Fig. 4). However, the current study did not provide a direct link between these two events and further research is needed to clarify this issue. Spindle–chromosome exchange experimentation revealed that such an epigenetic change was largely dependent on the cytoplasmic factors in Polg^m^ oocytes (Fig. 5). Moreover, metabolomic analysis clearly showed a reduction in the αKG level in oocytes from Polg^m^ mice (Fig. 6). Furthermore, we found that αKG supplement partly restored both the DNA methylation status and gene expression pattern in Polg^m^ embryos, concomitantly preventing the growth retardation of fetuses and placentas derived from Polg^m^ oocytes. Nonetheless, no significant difference in the expression of genes encoding the relevant enzymes was detected (Supplementary Figs S5–S7). Post-translational modifications may be involved in the activity control of these enzymes. For example, DNMT1 protein stability is closely associated with its acetylation and ubiquitination [57].

That environmental exposures in early life shape lifecycle health has been conceptualized as the Developmental Origin of Health and Disease (DOHaD). Mitochondria are crucial in providing intermediate metabolites necessary to modify epigenetic marks, which in turn can integrate environmental stimuli to fine-tune gene expression and affect later health. It has been reported that mtDNA copy number reduction in the embryo causes hypermethylation of Pparα in fetal liver, leading to impaired hepatic lipid metabolism in adult mice [58]. In combination with our data, these observations suggest that mitochondria genome may act as a receiver and integrator that links environmental exposures in early life to health in adults. Deciphering the mitochondrial function in oocytes may contribute to understanding the DOHaD theory.

Collectively, all these findings strongly support a model in which an increase in mtDNA mutation frequency in oocytes results in metabolic dysfunction, which, in turn, disrupts the genome methylation landscape and embryonic gene expression, consequently contributing to the developmental abnormalities of post-implantation embryos (Supplementary Fig. S9). Maternal obesity and aging have significant effects on the reproductive outcome. mtDNA mutation and mitochondrial dysfunction have been widely reported in germ cells from these females [59,60]. The present study demonstrates that metabolic dysfunction contributes to the reproductive disorders experienced by obese/old females and specifically implicates αKG as a promising therapeutic target for treatment of these conditions. Meanwhile, mitochondria fulfill a wide range of functions in diverse cellular processes; hence, this study cannot rule out the possibility that other pathways may simultaneously mediate the epigenetic changes observed in Polg^m^ oocytes/zygotes. For example, αKG is also an essential cofactor of the histone demethylases [55]. Erasing or remodeling of inherited histone modifications from oocytes and sperm in zygotes is necessary for subsequent embryo development [61]. Future work is needed to decipher the potential diverse effects of mtDNA mutations in oocytes on female reproduction.

## METHODS

Detailed materials and methods are available in the Supplementary data.

## DATA AVAILABILITY

The BS-Seq data and RNA-Seq data have been deposited in NCBI’s Gene Expression Omnibus and are accessible through GEO series accession number GSE149053 and code ‘ehuvuiamnpyztcj’.

## Supplementary Material

nwac136_Supplemental_files
