# Supplementary material for: Increased mtDNA mutation frequency in oocytes causes epigenetic alterations and embryonic defects
Source: Natl Sci Rev. 2022 Jul 13;9(10):nwac136. doi: 10.1093/nsr/nwac136 (PMC9616472; doi:10.1093/nsr/nwac136)
Supplement: nwac136_Supplemental_files [file nwac136_supplemental_files.zip › Supplemental_Tables_1-6.pdf]

Supplemental Table 1

Primer sequences for genotyping

	Primer sequence
Polg F	5′ –ATG TGG CCC AGG CTG TAA CT– 3′
Polg R	5′ –CTC GCT TTC TCC GTG ACT G– 3′

Primer sequences of genes for Bisulfite sequencing

Gene	***Primer sequence***
LINE-1	OF: 5′ –GTTAGAGAATTTGATAGTTTTGGAATAGG– 3′
	OR: 5′ –CCAAAACAAAACCTTTCTCAAACACTATAT– 3′
	IF: 5′ –TAGGAAATTAGTTTGAATAGGTGAGAGGT– 3′
	IR: 5′ –TCAAACACTATATTACTTTAACAATTCCCA– 3′
Wnt4	OF: 5′ –ATATATATGYGGGTGGAGTG– 3′
	OR/IR: 5′ –CCAAATCCTCATCTATATATAACTTAA – 3′
	IF: 5’ –TTTTAGGGAAGGATTAGAGTATG– 3’
Ahcy	OF: 5’ –TTTTAGGGAAGGATTAGAGTATG– 3’
	OR: 5’ –TATTTACCCAAAACCTACACAA– 3’
	IF: 5’ –GTTAGGGAAGTAGGTTTTAGTT– 3’
	IR: 5’ –CACTAAATCCCCAACAACAT– 3’
Anapc5	OF: 5’ –TGTTTTTATTTTTTTTTGTAAGGA– 3’
	OR: 5’ –AATTCTAAAAACCAAACCCAAT– 3’
	IF: 5’ –ATTTGTAGGTAGTTAYGTGTTAG– 3’
	IR: 5’ –CCCAATTTCTCTACAAAAACAAC– 3’
Phgdh	OF: 5’ –GGGGGTTGATTTTTAGTTTT– 3’
	OR: 5’ –CTAAACAAAATCTCTAATAACCTAAAA– 3’
	IF: 5’ –GGAAGAAGAAGAAGAAGAAG– 3’
	IR: 5’ –AACCTAAAATAACCTAAAATCAATTTT– 3’
Spns1	OF: 5’ –AATGGTAGAAGAGGATTGTTTG– 3’
	OR: 5’ –ATCCCAACATTCTAAAAACAAA– 3’
	IF: 5’ –GGATTGTTTGTTATAAGTTGTT– 3’
	IR: 5’ –CCTCCTAACCTTCAATCC– 3’
Xylb	OF: 5’ –GTTGATTTTAGGGTATAGGTTAG– 3’
	OR: 5’ –AACAAAAACACTTATCACAAAAA– 3’
	IF: 5’ –GGGTATAGGTTAGATAAAAGTAAT– 3’
	IR: 5’ –CAAATTCTAAATCAACCAAAACTAC– 3’

OF: outer forward; OR: outer reverse; IF: inner forward; IR: inner reverse

Primer sequences for quantification of mtDNA copy number

	Primer sequence
B6-F	5′ –AACCTGGCACTGAGTCACCA– 3
B6-R	5′ –GGGTCTGAGTGTATATATCATGAAGAGAAT – 3′

Primer sequences for detection of mtDNA mutation

Gene	*Primer sequence*
MTC	F: 5' –GCCAACTAGCCTCCATCTCATACTT – 3'
	R: 5' –GGGCGGGTTGTTGGTTTCAC – 3'
mt5495	F: 5' –TATCGTAACTGCCCATGCTTTTGT– 3'
	R: 5' –AGTTGTGTTTAGGTTGCGGTCTGT – 3'

Primer sequences for real-time RT-PCR

Gene	*Primer sequence*
Tet3	F: 5' –TGCGATTGTGTCGAACAAATAGT – 3'
	R: 5' –TCCATACCGATCCTCCATGAG – 3'
Dnmt1	F: 5' –AAGAATGGTGTTGTCTACCGAC – 3'
	R: 5' –CATCCAGGTTGCTCCCCTTG – 3'
Dnmt3a	F: 5' –GAGGGAACTGAGACCCCAC – 3'
	R: 5' –CTGGAAGGTGAGTCTTGGCA – 3'
Dnmt3b	F: 5' –CGTTAATGGGAACTTCAGTGACC – 3'
	R: 5' –CTGCGTGTAATTCAGAAGGCT – 3'
Gapdh	F: 5' –AGGTCGGTGTGAACGGATTTG – 3'
	R: 5' –TGTAGACCATGTAGTTGAGGTCA – 3'
Ms	F: 5' –AGGCCACTGAAAGGCAACAA– 3'
	R: 5' –GTTCATCCGGTAGGCCAAGT– 3'
Mat	F: 5' –GTGCTGGATGCTCACCTCAAG– 3'
	R: 5' –CCACCCGCTGGTAATCAACC– 3'
Ahcy	F: 5' –CCCTACAAAGTCGCGGACATC– 3'
	R: 5' –GAGGCTGAGTACATCTCCCG– 3'
Shmt1	F: 5' –CAGGGCTCTGTCTGATGCAC– 3'
	R: 5' –CGTAACGCGCTCTTGTCAC– 3'
Shmt2	F: 5' –TGGCAAGAGATACTACGGAGG– 3'
	R: 5' –GCAGGTCCAACCCCATGAT– 3'
Mthfr	F: 5' –CTGGGCACTGTTATCCATCCC– 3'
	R: 5' –TCCTGCTGATAGAGGGTGGC– 3'
Idh2	F: 5' –GGAGAAGCCGGTAGTGGAGAT– 3'
	R: 5' –GGTCTGGTCACGGTTTGGAA– 3'
Idh3	F: 5' –TGGGTGTCCAAGGTCTCTC– 3'
	R: 5' –CTCCCACTGAATAGGTGCTTTG– 3'
α-Kgdh	F: 5' –GTTTCTTCAAACGTGGGGTTCT– 3'
	R: 5' –GCATGATTCCAGGGGTCTCAAA– 3'
Scs	F: 5' –ACCCTTTCGCTGCATGAATAC– 3'
	R: 5' –CCTGTGCCTTTATCACAACATCC– 3'
Sdh	F: 5' –GGAACACTCCAAAAACAGACCT– 3'
	R: 5' –CCACCACTGGGTATTGAGTAGAA– 3'

Phgdh	F: 5’ –ATGGCCTTCGCAAATCTGC– 3’
	R: 5’ –AGTTCAGCTATCAGCTCCTCC– 3’
Xylb	F: 5’ –TCAGCACGCAGCAGGTTAAA– 3’
	R: 5’ –CGCCCTGAGTTCCAAATTCC– 3’
Anapc5	F: 5’ –TCAGCACGCAGCAGGTTAAA– 3’
	R: 5’ –GCGAGGTACGGTAACCCAAA– 3’
Spns1	F: 5’ –CCAAGCAGATGATCCTGATGAC– 3’
	R: 5’ –TGTAGCCCCTCACAGTCTGG– 3’

Supplemental Table 2

149 differentially methylated 20-kbp regions identified between WT and Polg^{m} oocytes, related to Fig. 3D-E

chr	start	end	qvalue	difference	cpg numbe	WT	PolgM	gene
chr1	24600000	24620000	3.06E-06	11.29361	153	56.661	45.367	.
chr1	1.36E+08	1.36E+08	3.74E-08	21.848253	145	56.418	34.569	Gm4793,Nav1
chr1	1.81E+08	1.81E+08	1.67E-06	21.061954	141	35.82	14.758	Pycr2
chr1	1.9E+08	1.9E+08	5.25E-05	25.032905	121	69.643	44.61	Smyd2
chr10	8240000	8260000	0.0011493	-34.290123	27	48.92	83.21	Ust
chr10	47060000	47080000	0.02453	26.111111	10	34.444	8.3333	.
chr10	58220000	58240000	7.92E-05	6.132867	273	12.702	6.5695	Dux,Gm4981
chr10	1.16E+08	1.16E+08	0.0024438	39.372428	27	78.323	38.951	.
chr11	5980000	6000000	1.90E-07	-23.773487	157	38.642	62.415	Camk2b
chr11	83840000	83860000	5.36E-06	19.412111	81	36.73	17.318	Hnf1b
chr11	1.07E+08	1.07E+08	0.0095434	27.95306	51	51.374	23.42	Pitpnc1
chr11	1.09E+08	1.09E+08	3.51E-07	-7.132493	91	29.707	36.84	.
chr11	1.2E+08	1.2E+08	0.030735	7.423399	246	11.485	4.0612	Bahcc1
chr11	1.2E+08	1.2E+08	0.045975	6.806842	288	12.534	5.7274	Bahcc1
chr12	8520000	8540000	0.03612	-21.476217	45	56.746	78.222	Slc7a15
chr12	20220000	20240000	0.0002154	7.200461	456	48.854	41.653	.
chr12	1.12E+08	1.12E+08	1.10E-07	9.915749	415	18.212	8.296	Kif26a
chr13	44860000	44880000	4.62E-06	-10.173468	71	31.027	41.201	Jarid2
chr13	49420000	49440000	0.0030976	-26.372155	83	63.068	89.44	Ippk
chr13	51620000	51640000	0.00025273	38.02005	38	51.734	13.713	.
chr13	58040000	58060000	0.011307	26.284722	64	82.721	56.436	Klhl3
chr13	58080000	58100000	1.27E-12	34.594416	74	94.111	59.516	Klhl3
chr13	1.01E+08	1.01E+08	0.0026564	33.095238	31	47.199	14.104	.
chr13	1.19E+08	1.2E+08	0.0010588	-10.453227	192	45.263	55.717	3110070M22Rik, 4833420G17Rik, Tmem267
chr14	8240000	8260000	1.81E-05	29.106807	59	78.079	48.972	Acox2
chr14	19400000	19420000	0.014917	4.895088	171	28.694	23.799	.
chr14	48160000	48180000	0.043251	-13.925307	77	15.32	29.246	Peli2
chr14	55060000	55080000	1.23E-12	19.297127	278	43.345	24.048	Zfhx2,Zfhx2os
chr14	58880000	58900000	0.042558	22.008022	31	23.782	1.7742	.
chr14	60680000	60700000	0.00044073	15.535767	125	34.426	18.89	Spata13
chr14	60740000	60760000	0.043958	21.735857	78	75.171	53.435	Spata13
chr14	1.03E+08	1.03E+08	0.036769	6.051587	52	26.885	20.833	Fbxl3
chr15	27700000	27720000	1.22E-08	30.669092	72	45.623	14.954	.
chr15	28160000	28180000	0.00049341	24.070388	61	38.778	14.707	.
chr15	76160000	76180000	3.35E-07	12.751944	262	81.818	69.066	Plec
chr15	76200000	76220000	9.79E-05	18.214064	216	67.183	48.969	Mir1942,Plec
chr15	76300000	76320000	1.91E-05	19.907414	172	48.429	28.521	Oplah
chr15	80580000	80600000	0.016947	26.050852	45	47.34	21.289	Grap2
chr15	83600000	83620000	0.0022564	18.484288	148	71.603	53.119	Scube1
chr15	98720000	98740000	0.0041273	-18.64513	128	34.909	53.554	Arf3,Ccdc65,Fkbp11
chr15	99420000	99440000	0.00077487	10.757284	231	15.103	4.3457	Nckap5l
chr16	11140000	11160000	2.83E-43	-7.840702	104	14.046	21.887	Zc3h7a
chr16	57380000	57400000	9.07E-26	-6.61391	98	4.6524	11.266	Cmss1,Filip1l
chr17	6740000	6760000	1.92E-16	17.246454	238	27.105	9.8588	Ezr
chr17	35000000	35020000	1.34E-05	13.227091	353	31.084	17.857	D17H6S56E-5,Vars,Vwa7
chr17	39840000	39860000	0	-11.884899	1201	3.7926	15.678	Rn45s
chr18	38260000	38280000	0.00026999	18.131478	73	45.78	27.648	Dele1,Pcdh12
chr18	51000000	51020000	0.0003985	13.052071	49	24.087	11.035	.
chr18	53460000	53480000	0.004534	12.637159	195	25.613	12.976	Prdm6

chr18	60600000	60620000	0.011959	10.883096	169	57.576	46.693	Synpo
chr18	61700000	61720000	0.0019592	-15.089186	60	77.429	92.519	Grpel2,Pcyox1l
chr18	65080000	65100000	5.08E-18	40.679344	87	50.806	10.127	Nedd4l
chr18	65140000	65160000	0.02854	28.359788	63	74.727	46.367	Nedd4l
chr18	68680000	68700000	5.95E-06	-7.903914	46	4.7572	12.661	.
chr19	6820000	6840000	8.56E-07	14.938071	202	35.016	20.078	Mir5046,Rps6ka
chr19	6840000	6860000	0.0002999	11.291687	278	64.267	52.976	Ccdc88b,Rps6ka
chr19	25380000	25400000	0.03972	23.631687	54	74.578	50.947	Kank1
chr19	61180000	61200000	2.23E-06	-9.48203	42	4.4052	13.887	.
chr2	5360000	5380000	8.68E-05	-9.303997	33	25.32	34.624	Camk1d,Mir466d
chr2	24660000	24680000	0.003762	19.251435	87	47.236	27.984	Cacna1b
chr2	26460000	26480000	6.50E-05	8.201862	359	14.506	6.3041	Mir6996,Notch1
chr2	31080000	31100000	0.029719	25.854915	57	69.951	44.096	Fnbp1
chr2	31120000	31140000	0.001994	22.352092	77	38.737	16.385	Fnbp1
chr2	81140000	81160000	0.0092686	14.831507	42	26.234	11.402	.
chr2	98660000	98680000	0.00012444	4.792857	133	28.565	23.772	.
chr2	1.02E+08	1.02E+08	0.0029854	26.236552	60	60.666	34.429	Ldlrad3
chr2	1.02E+08	1.02E+08	0.0055792	22.579127	84	68.57	45.991	Ldlrad3
chr2	1.12E+08	1.12E+08	3.03E-05	43.271605	36	75.88	32.608	Emc7
chr2	1.3E+08	1.3E+08	5.30E-06	26.75684	85	54.38	27.623	Ebf4
chr2	1.59E+08	1.59E+08	7.95E-07	30.144966	74	44.797	14.652	Ppp1r16b
chr2	1.59E+08	1.59E+08	2.37E-06	30.697479	85	45.683	14.986	Ppp1r16b
chr2	1.59E+08	1.59E+08	2.38E-05	24.239826	81	49.947	25.708	Ppp1r16b
chr2	1.63E+08	1.63E+08	0.011849	20.30697	87	51.431	31.124	Tox2
chr2	1.68E+08	1.68E+08	0.00013582	19.834757	149	66.891	47.056	Tmem189
chr2	1.75E+08	1.75E+08	0.00077214	19.476252	102	68.352	48.876	.
chr3	5860000	5880000	4.44E-37	-11.094625	85	4.4161	15.511	.
chr3	41100000	41120000	0.015784	35.126396	18	52.41	17.284	.
chr3	1.18E+08	1.18E+08	0.012945	14.346864	122	19.379	5.0319	Plppr5
chr3	1.22E+08	1.22E+08	0.0024246	23.59831	62	48.105	24.507	Slc44a3
chr4	1.09E+08	1.09E+08	0.036233	32.05873	25	92.565	60.506	Eps15
chr4	1.22E+08	1.22E+08	0.045481	16.441534	146	44.227	27.786	.
chr4	1.27E+08	1.27E+08	0.0078239	22.210579	63	56.99	34.78	.
chr4	1.29E+08	1.29E+08	0.013993	20.370821	88	72.494	52.123	Csmd2
chr4	1.32E+08	1.32E+08	0.001608	14.143236	132	88.268	74.125	Ptpru
chr4	1.35E+08	1.35E+08	0.043381	17.431725	99	42.186	24.755	.
chr4	1.37E+08	1.37E+08	0.03025	11.250339	174	41.157	29.907	Wnt4
chr4	1.38E+08	1.38E+08	0.0083666	7.033155	333	9.2672	2.2341	Hspg2
chr4	1.49E+08	1.49E+08	0.023613	-11.280155	246	42.935	54.215	Casz1,Gm13205
chr4	1.5E+08	1.5E+08	1.01E-05	25.746978	123	70.614	44.867	Spsb1
chr4	1.5E+08	1.5E+08	0.0049943	10.41237	177	14.062	3.65	Gpr157,Slc2a5
chr4	1.52E+08	1.53E+08	0.00556	17.152822	192	83.578	66.425	Nphp4
chr4	1.53E+08	1.53E+08	3.39E-09	23.088172	194	84.513	61.425	Nphp4
chr4	1.53E+08	1.53E+08	1.31E-11	23.430097	241	72.708	49.278	Nphp4
chr4	1.55E+08	1.55E+08	0.0046145	8.53617	261	14.413	5.8766	Mmel1,Prxl2b
chr5	24960000	24980000	6.66E-05	31.504486	69	65.132	33.628	Prkag2
chr5	24980000	25000000	1.32E-07	36.849876	77	69.271	32.421	Prkag2
chr5	34300000	34320000	0.043526	23.248509	63	56.48	33.232	Cfap99
chr5	72580000	72600000	0.0029502	17.397292	119	24.458	7.0612	Slc10a4l,Zar1
chr5	1.12E+08	1.12E+08	4.79E-05	18.368024	145	83.147	64.779	Tpst2
chr5	1.14E+08	1.14E+08	0.018112	9.138161	200	13.197	4.0591	Mvk
chr5	1.21E+08	1.21E+08	0.010701	11.103502	205	22.429	11.325	Rasal1
chr5	1.22E+08	1.22E+08	0.037342	17.209724	124	67.243	50.033	Cux2
chr5	1.44E+08	1.44E+08	0.00013276	27.077645	59	47.679	20.602	.
chr5	1.46E+08	1.46E+08	1.36E-15	-5.471094	88	20.507	25.978	Cdk8
chr6	3200000	3220000	2.52E-08	-1.203694	82	21.08	22.283	.
chr6	99520000	99540000	0.041823	24.297201	76	51.718	27.421	Foxp1
chr6	1.16E+08	1.16E+08	5.87E-07	8.769748	258	22.015	13.245	Plxnd1

chr6	1.36E+08	1.36E+08	0.03042	44.246032	20	73.889	29.643	Grin2b
chr6	1.45E+08	1.45E+08	0.021279	34.70679	36	50.309	15.602	Lrmp
chr6	1.45E+08	1.45E+08	2.34E-05	27.541521	87	44.19	16.649	Lrmp
chr7	30280000	30300000	0.00014943	21.343754	148	35.908	14.564	Clip3,Thap8,Wdr62
chr7	33900000	33920000	0.0095412	25.672336	35	38.672	13	.
chr7	49140000	49160000	0.00014529	47.848609	31	76.983	29.135	9130015G15Rik,Nav2
chr7	49200000	49220000	0.031676	24.738569	69	59.415	34.677	Nav2
chr7	64440000	64460000	0.0048148	38.508498	27	55.155	16.646	.
chr7	67840000	67860000	1.79E-05	23.180539	63	44.833	21.653	.
chr7	78900000	78920000	4.30E-12	-30.33661	107	54.968	85.305	Aen,Isg20
chr7	78920000	78940000	3.29E-06	-28.151314	79	21.262	49.413	Isg20
chr7	79620000	79640000	5.33E-13	-63.528634	34	32.622	96.151	.
chr7	80040000	80060000	2.34E-12	32.173289	109	39.337	7.1638	Zfp710
chr7	80060000	80080000	3.15E-28	54.332933	86	66.213	11.88	Gm21057,Zfp71
chr7	80080000	80100000	2.30E-14	32.679094	152	56.91	24.231	Idh2,Zfp710
chr7	80360000	80380000	1.67E-08	13.79644	276	17.706	3.9093	Fes,Man2a2
chr7	96880000	96900000	0.033339	19.658593	58	26.79	7.1312	Tenm4
chr7	99900000	99920000	0.015293	15.951746	108	58.618	42.666	Xrra1
chr7	1.01E+08	1.01E+08	0.0034637	11.272876	147	31.147	19.874	Arap1
chr7	1.01E+08	1.01E+08	2.32E-05	12.576103	221	29.943	17.367	Arap1
chr7	1.13E+08	1.13E+08	3.77E-05	33.480392	51	81.768	48.287	Tead1
chr7	1.17E+08	1.17E+08	0.044147	18.138736	52	39.193	21.054	.
chr7	1.21E+08	1.21E+08	8.42E-05	26.131166	93	55.956	29.825	Eef2k
chr7	1.22E+08	1.22E+08	0.022167	-27.153604	48	22.294	49.448	Prkcb
chr7	1.4E+08	1.4E+08	0.020885	7.251652	140	12.094	4.8425	Cd163l1
chr7	1.41E+08	1.41E+08	0.0013277	16.407116	95	28.37	11.963	B230206H07Rik,Eps8l2
chr7	1.42E+08	1.42E+08	0.014887	11.364666	158	19.437	8.0724	Muc6
chr7	1.44E+08	1.44E+08	4.40E-05	17.880659	142	40.232	22.351	Osbpl5
chr8	35760000	35780000	6.27E-06	28.922902	42	93.074	64.152	Gm19410
chr8	71600000	71620000	0.017814	13.854125	199	47.351	33.496	Colgalt1,Fam129
chr8	75560000	75580000	0.01769	31.655358	35	63.754	32.098	Iqcm
chr8	84960000	84980000	1.73E-10	11.506992	266	28.622	17.115	Junb,Prdx2,Rnaseh2a
chr8	87660000	87680000	0.0044207	14.009724	111	52.663	38.654	Zfp423
chr8	1.22E+08	1.22E+08	0.0064917	17.549954	144	67.933	50.383	Jph3
chr9	3020000	3040000	8.69E-11	5.6601	566	26.534	20.874	Mir101c
chr9	42560000	42580000	4.62E-07	38.70416	58	69.989	31.285	Grik4
chr9	42580000	42600000	0.0057239	28.607373	46	87.522	58.915	Grik4
chr9	42600000	42620000	0.040823	28.890542	48	50.789	21.898	Grik4
chr9	65260000	65280000	0.011727	17.990786	87	26.985	8.9943	Cilp
chr9	77140000	77160000	0.0084108	28.898095	26	59.04	30.141	Mlip
chr9	1.08E+08	1.08E+08	0.00020071	12.783111	271	36.263	23.48	Sema3f
chr9	1.08E+08	1.08E+08	0.0145	-10.064198	381	20.234	30.298	Bsn

Supplemental Table 3

Hypo- and hyper-methylated domains identified between WT and Polg^{m} oocytes, related to Fig. 3H

3 hyper-methylated domains

chr	start	end	qvalue	methylation difference	CpG numbe	Polg^{m}	WT	overlapped genes
chr15	83791530	83809187	0.0089811	-14.751434	132	67.443	82.195	Mpped1
chr4	1.31E+08	1.31E+08	0.0047817	-10.44685	250	76.259	86.706	Matn1
chr8	84743879	84771937	0.012662	-7.225384	170	83.041	90.267	Nfix

57 hypo-methylated domains

chr	start	end	qvalue	hylation differe	G numb	Polg^{m}	WT	overlapped genes
chr10	60933991	61034044	0.013529	-7.027024	585	3.0235	10.051	Gm20611,Mir466j,Pldi
chr11	35403000	35685371	6.32E-05	-7.465078	960	10.91	18.375	Mir218-2,Slit3
chr11	51443000	51567125	0.0017746	-7.31556	576	10.518	17.833	Col23a1
chr11	59045092	59134387	4.40E-06	-6.66714	820	4.2189	10.886	Obscn
chr11	94392957	94472100	0.0091996	-5.296274	698	3.5306	8.8269	Abcc3,Cacna1
chr11	1.14E+08	1.14E+08	0.043851	-5.817922	583	2.2594	8.0773	Sdk2
chr11	1.17E+08	1.17E+08	0.0068541	-5.663736	765	2.7263	8.3901	Septin9
chr11	1.19E+08	1.19E+08	0.0016758	-5.075279	1042	2.2759	7.3512	Mir6934,Tbc1d16
chr11	1.2E+08	1.2E+08	0.044966	-5.917107	706	5.3038	11.221	Bahcc1
chr12	76579991	76653001	0.0045489	-6.130285	670	3.3189	9.4492	Sptb
chr12	1.05E+08	1.05E+08	0.00050611	-7.043017	680	4.1408	11.184	Clmn
chr12	1.12E+08	1.12E+08	2.51E-05	-5.577143	936	2.5282	8.1054	.
chr15	86610492	86860263	5.29E-05	-6.491107	857	4.9789	11.47	.
chr15	86924338	87499898	2.29E-05	-5.254765	1430	5.9272	11.182	A930027H12Rik
chr15	97971469	97999660	9.98E-06	-9.959578	418	6.3006	16.26	Col2a1
chr16	34773000	35015484	1.44E-05	-6.481593	988	3.183	9.6646	1700119H24Rik,E130310I04Rik,Mylk
chr17	47141332	47323379	8.44E-05	-5.258019	1083	2.9511	8.2092	Trerf1
chr17	83918905	84014649	0.0058418	-6.146551	606	6.4618	12.608	1810073O08Rik,8430430B14Rik
chr17	86418758	86753009	0.013261	-5.021389	1243	4.5174	9.5388	Prkce
chr19	6430767	6497095	0.0045069	-6.001436	756	3.0946	9.096	Nrxn2
chr2	26462360	26501822	0.0019159	-6.965671	531	5.0072	11.973	Mir6996,Notch
chr2	27888425	28042316	0.0020077	-4.739488	1021	3.5154	8.2549	Col5a1
chr2	31354774	31425245	0.032422	-5.48818	648	3.4967	8.9849	Hmcn2
chr2	1.57E+08	1.57E+08	0.008483	-7.288707	256	3.004	10.293	Src
chr2	1.66E+08	1.66E+08	9.64E-05	-7.762291	480	3.5088	11.271	.
chr2	1.66E+08	1.66E+08	0.0347	-7.146832	497	6.3807	13.528	Gm11468
chr2	1.68E+08	1.68E+08	0.00027625	-6.058804	891	4.7133	10.772	A530013C23Rik
chr2	1.74E+08	1.74E+08	0.00011038	-8.384054	378	2.1044	10.488	Ankrd60
chr2	1.75E+08	1.75E+08	0.0097138	-6.199746	676	3.0108	9.2105	Zfp831
chr3	51701839	51929001	0.013478	-5.914137	784	2.8188	8.733	Maml3
chr4	46664974	46846136	6.20E-05	-5.034845	1197	3.5555	8.5904	Gabbr2
chr4	63216309	63357848	9.55E-07	-6.412458	908	6.9026	13.315	Col27a1,Mir455,Orm1,Orm3
chr4	1.07E+08	1.07E+08	6.76E-05	-6.665639	544	1.7935	8.4592	Glis1

chr4	1.25E+08	1.26E+08	4.19E-14	-6.12551	1863	2.5094	8.6349	Grik3,Mir692-2,Mir692-3
chr4	1.28E+08	1.28E+08	1.24E-05	-5.379398	1443	3.4649	8.8443	Csmd2,Hmgb4,Hmgb4os
chr4	1.31E+08	1.31E+08	0.0047183	-8.757787	370	3.5028	12.261	.
chr4	1.37E+08	1.38E+08	3.26E-10	-5.85547	1267	2.0574	7.9128	Hspg2,Mir701
chr4	1.4E+08	1.41E+08	2.52E-07	-5.200955	2062	4.3813	9.5823	Gm13016,Gm9867
chr4	1.55E+08	1.55E+08	0.00031847	-5.461411	871	3.9844	9.4459	Gm13133,Gm52688,Prdm16
chr5	30364767	30465928	1.46E-05	-7.100185	737	3.1536	10.254	Drc1,Otof
chr5	1.29E+08	1.29E+08	0.049051	-5.676827	953	7.3985	13.075	Adgrd1
chr6	91979650	92079099	0.015954	-5.969379	637	3.7886	9.758	Fgd5
chr6	1.14E+08	1.14E+08	0.0077127	-6.00189	615	3.5543	9.5562	Slc6a11
chr6	1.14E+08	1.15E+08	0.001627	-4.998128	906	2.6921	7.6902	Hrh1
chr6	1.19E+08	1.19E+08	2.07E-10	-6.411519	1540	3.5078	9.9193	4931430N09Rik,Cacna1c
chr7	35121288	35184994	0.0039424	-6.519967	431	2.0377	8.5577	Cebpa
chr7	44630752	44633377	0.017722	-26.34765	51	10.86	37.207	Myh14
chr7	66173487	66386350	0.00097275	-5.570154	906	5.7004	11.271	Chsy1,Lrrk1,Mir7057
chr7	1.01E+08	1.01E+08	0.037494	-7.404203	380	8.652	16.056	Arhgef17,Mir3102
chr7	1.11E+08	1.12E+08	9.20E-06	-7.050743	826	5.1231	12.174	Galnt18
chr7	1.17E+08	1.18E+08	0.015602	-6.327651	645	3.7525	10.08	Xylt1
chr7	1.36E+08	1.37E+08	2.04E-05	-5.60654	1204	4.0492	9.6558	C030029H02Rik,C230079O03Rik,Gm36849,Gm6249
chr8	19685286	19758195	0.048235	-8.261801	231	10.148	18.41	4930467E23Rik,Gm21119
chr8	1.17E+08	1.17E+08	0.0018487	-4.23342	1527	3.7851	8.0185	Cmip
chr8	1.19E+08	1.19E+08	7.64E-08	-8.056711	734	5.5454	13.602	Cdh13
chr8	1.21E+08	1.21E+08	0.0098062	-6.312074	624	2.7306	9.0426	.
chr9	48675248	48834236	0.00065747	-5.861823	847	2.8864	8.7482	Zbtb16

Supplemental Table 4

Identified 87 DMRs between WT and Polg^{m} mice, related to Fig. S3

chr	start	end	associated gene	p-value	difference in methylation level	CpG sites	mean value in WT oocyte	mean value in D257A oocyte
chr13	49421000	49421210	*Ippk*	0.0066	-75.940171	13	7.4786	83.419
chr10	80315047	80315315	*Grin3b,Hcn2,Kiss1l*	3.00E-05	-50.536449	27	20.122	70.658
chr1	133386270	133386933	*Sox13*	0.016	-49.603175	21	33.201	82.804
chr8	10154045	10154300	*Myo16*	7.50E-05	-48.349298	29	12.628	60.977
chr4	153495159	153495580	*Ajap1*	0.016	-46.531085	16	8.2986	54.83
chr2	181088361	181088791	*Kcnq2*	0.022	-46.127278	18	28.331	74.458
chr18	61707503	61707984	*Pcyox1l*	6.20E-08	-34.107655	27	59.226	93.333
chr7	126949928	126950007	*Asphd1*	0.026	-29.378307	21	69.392	98.77
chr7	45715207	45715448	*Dbp,Sphk2*	0.021	-25.136345	26	70.505	95.641
chr19	61199680	61199780	*Gm7102*	3.10E-09	-17.31611	19	1.9085	19.225
chr17	39843000	39843464	*Rn45s*	4.30E-58	-17.04099	79	3.6706	20.712
chr17	39843561	39844049	*Rn45s*	3.20E-58	-16.631536	88	4.0085	20.64
chr13	44869718	44870021	*Jarid2*	0.0092	-16.242096	26	5.9904	22.233
chr3	5860679	5860765	*Pex2*	4.40E-15	-14.729078	26	2.701	17.43
chr17	39846233	39847392	*Rn45s*	2.20E-104	-14.130191	169	3.6424	17.773
chr9	123461889	123462107	*Lars2*	2.90E-10	-13.813235	22	4.7447	18.558
chr18	68692071	68692185	*Mir6356*	4.10E-08	-13.651181	20	3.4679	17.119
chr17	39844320	39844389	*Rn45s*	3.60E-10	-13.378238	20	4.7195	18.098
chr17	39845444	39845646	*Rn45s*	2.30E-28	-12.769891	53	3.9863	16.756
chr17	39844049	39844202	*Rn45s*	1.40E-14	-11.851908	28	3.4459	15.298
chr17	39847398	39848188	*Rn45s*	1.60E-66	-11.673634	118	3.7861	15.46
chr3	5860339	5860675	*Pex2*	9.30E-23	-11.595065	47	3.7467	15.342
chr17	39846216	39846231	*Rn45s*	0.019	-11.574626	10	3.2951	14.87
chr17	39848405	39848823	*Rn45s*	5.60E-69	-11.231507	119	3.5348	14.766
chr2	5379130	5379355	*Camk1d,Mir466d*	8.00E-05	-11.036831	25	7.7783	18.815
chr16	57391421	57391657	*Cmss1,Filip1l*	3.90E-36	-10.30076	59	3.0492	13.35
chr17	39845732	39846204	*Rn45s*	7.20E-75	-9.739264	131	3.1019	12.841
chr17	39844389	39845434	*Rn45s*	1.20E-122	-9.119755	247	3.3979	12.518
chr6	3201374	3201587	*Gm8579*	4.10E-09	-9.028088	40	3.4303	12.458
chr17	39848202	39848306	*Rn45s*	1.80E-12	-8.944139	33	3.7757	12.72
chr16	11143974	11144309	*Zc3h7a*	1.10E-43	-8.499279	93	4.1485	12.648
chr17	39844202	39844311	*Rn45s*	1.50E-10	-8.236116	31	4.3434	12.58
chr11	109011736	109012100	*Axin2*	2.50E-11	-7.820854	58	6.802	14.623
chr5	146260996	146261256	*Cdk8*	4.20E-14	-7.214722	42	2.8429	10.058
chr17	39845646	39845728	*Rn45s*	2.80E-08	-6.844212	21	2.9902	9.8344
chrM	3140	4440		0.018	8.764385	56	64.625	55.861
chrM	5588	6581		1.90E-07	13.577418	37	57.105	43.528
chr17	27077023	27077795	*Itpr3,Mir7677*	0.0056	33.400794	20	94.472	61.071
chr10	94128931	94129018	*Fgd6*	0.00047	34.583333	14	38.552	3.9683
chr19	36918979	36919084	*Fgfbp3*	0.0056	35.34709	25	40.572	5.2254
chr4	152494621	152495203	*Nphp4*	0.032	35.720681	22	50.998	15.278
chrY	90807458	90807533	*Erdr1*	0.042	36.220185	10	59.018	22.798
chr4	10888680	10889371	*2610301B20Rik*	0.049	36.550558	27	51.371	14.821
chr18	36993998	36994321	*b21,Pcdhb22,Pcdh*	0.041	37.671958	15	40.265	2.5926
chr8	84978471	84978896	*Junb*	6.20E-12	39.15733	34	45.49	6.3329
chr18	75695417	75696069	*Ctif,Gm10532*	0.0011	41.712522	30	60.567	18.855
chr11	88169894	88170055	*16O15Rik,Cuedc1,M*	0.024	42.046784	19	51.842	9.7953
chr17	6758030	6758182	*Ezr*	0.028	42.137897	16	49.082	6.9444
chr7	80362561	80363053	*Man2a2*	0.0096	42.197657	14	45.928	3.7302
chr4	137296350	137296484	*Wnt4*	0.026	42.574189	23	43.299	0.72464
chr7	98128427	98128537	*apn5,Gm16938,Om*	0.0084	42.781746	10	47.948	5.1667
chrX	85048394	85048949	*Dmd,Tsga8*	3.00E-05	43.675373	25	48.72	5.0444
chr10	126979932	126980072	*dsp2,Mir26a-2,Mir5*	0.009	44.518141	14	48.883	4.3651
chr7	67849064	67849505	*4833412C05Rik*	9.00E-10	44.750094	28	53.424	8.6739
chr2	31032801	31033248	*Fnbp1*	0.013	45.100529	15	56.37	11.27
chr7	80363235	80363468	*Man2a2*	0.016	45.103175	10	47.556	2.4524
chr1	180905630	180906556	*Pycr2*	1.10E-05	45.292362	39	67.567	22.275
chr4	152537017	152538046	*Nphp4*	0.016	45.647066	30	70.095	24.448
chr13	111687505	111687607	*Mier3*	0.017	46.198413	10	51.476	5.2778
chr17	26275999	26276584	*Luc7l*	0.029	47.132937	16	62.604	15.471
chr18	65084308	65084721	*Mir122,Mir466p,Ne*	0.024	48.325397	20	60.659	12.333
chr4	152512331	152512666	*Nphp4*	0.035	48.611111	10	50.278	1.6667
chr2	158695766	158696017	*Ppp1r16b*	0.035	49.065657	11	51.465	2.399
chr9	106460507	106460781	*Gpr62,Pcbp4*	0.016	49.322562	14	62.341	13.019
chr14	21857350	21857812	*57M04Rik,Comtd1,*	0.0039	50.640212	15	57.788	7.1481
chr2	130421644	130422641	*Pced1a,Vps16*	0.0032	50.991434	21	89.365	38.374
chr4	152538046	152538227	*Nphp4*	0.015	51.236572	12	51.742	0.50505

chr7	80081738	80082126	*Gm21057,Zfp710*	0.0028	53.923611	16	95.59	41.667
chr14	60639108	60640417	*Spata13*	0.0055	54.367063	20	72.875	18.508
chr18	65082998	65084276	*Mir122,Mir466p,Ne*	1.60E-09	54.480657	35	65.145	10.664
chr6	145141732	145142201	*Casc1,Lrmp*	0.00031	54.758424	19	60.293	5.5347
chr14	55074028	55074846	*Zfhx2,Zfhx2os*	8.20E-07	56.605568	21	62.709	6.1036
chr5	114458983	114459373	*Mvk*	3.00E-05	57.048942	20	82.243	25.194
chr7	80367411	80368355	*Man2a2*	0.00096	58.011696	19	69.035	11.023
chr13	51638003	51638244	*Cks2*	0.02	58.5	10	70.278	11.778
chr15	76174733	76175006	*Mir1942,Plec*	0.011	61.013072	17	75.98	14.967
chr2	112454912	112455225	*Emc7*	0.0047	61.527778	16	80.278	18.75
chr4	152546553	152547607	*Nphp4*	2.70E-12	63.121693	38	85.703	22.581
chr8	87662941	87663208	*Zfp423*	4.80E-06	64.814815	15	69.259	4.4444
chr8	121753123	121753542	*Jph3*	0.00013	69.632937	16	95.972	26.339
chr7	80057715	80058169	*Gm21057,Zfp710*	5.80E-11	70.288933	27	82.437	12.148
chr10	8807072	8807852	*Sash1*	6.80E-05	70.47619	16	88.438	17.961
chr17	35011828	35011911	*Vars*	0.042	70.833333	10	81.667	10.833
chr7	80078918	80079225	*Gm21057,Zfp710*	0.0079	71.888889	10	92.444	20.556
chr2	28533349	28533540	*Ralgds*	2.20E-05	74.074074	18	96.296	22.222
chr7	80081058	80081375	*Gm21057,Zfp710*	0.02	75.364265	13	92.501	17.137
chr11	102026962	102026996	*Mpp3*	0.00084	76.111111	10	90	13.889

Supplemental Table 5

Differentally expressed genes between WT and Polgm morula embryos, related to Fig. 4						
gene	baseMean	log2FoldChange (Polg^{m}/WT)	lfcSE	stat	pvalue	padj
Eif2d	4858.648768	-1.109918414	0.2578566	-4.3044	1.67E-05	0.01040149
Rnpep	2809.283532	-1.130566241	0.2880075	-3.92547	8.66E-05	0.0228122
Csrp1	901.2208792	-1.092083625	0.3111154	-3.51022	0.000448	0.04929059
Dhx9	984.9171294	1.549490603	0.4009958	3.864107	0.000111	0.02370727
AA467197	1032.937817	1.186092908	0.3020377	3.92697	8.60E-05	0.0228122
Necab3	121.0115017	-2.094378404	0.5305394	-3.94764	7.89E-05	0.0228122
Ahcy	363.6458356	-1.277791049	0.3564168	-3.5851	0.000337	0.0436758
Aurka	11207.98891	-1.039313891	0.2738089	-3.79576	0.000147	0.02633044
Sema4a	293.9095727	-2.46172003	0.4191775	-5.87274	4.29E-09	1.86E-05
Efna1	609.1592732	1.188226379	0.3333552	3.564445	0.000365	0.04466415
Tdpoz2	972.7057271	-1.265326403	0.3233191	-3.91355	9.09E-05	0.02326397
Phgdh	20925.64576	-1.150327039	0.3230625	-3.5607	0.00037	0.04467768
Cnn3	1340.917612	-1.219640998	0.2970987	-4.10517	4.04E-05	0.01952066
Rraga	223.9885321	-1.512246046	0.4189139	-3.60992	0.000306	0.04162199
Kti12	2391.666712	-1.051291049	0.273016	-3.85066	0.000118	0.02382598
Mpl	173.0412064	-1.611179999	0.4548332	-3.54235	0.000397	0.04636659
Fabp3	5877.759373	1.480611957	0.3031141	4.884669	1.04E-06	0.00150172
Ldlrap1	912.5942526	-1.132727669	0.3023509	-3.7464	0.000179	0.02908386
Cpeb2	1431.398965	1.116622314	0.2947591	3.788254	0.000152	0.0263884
Atp8a1	276.8337272	1.438436595	0.4028572	3.570587	0.000356	0.04454604
Anapc5	2259.972383	-1.268142782	0.2938113	-4.31618	1.59E-05	0.01040149
Tmem43	904.8697315	-1.426165216	0.3592914	-3.96938	7.21E-05	0.02238202
Wnk1	1652.958818	1.076636798	0.2811068	3.829992	0.000128	0.02476077
Cops7a	9369.609026	-1.009050495	0.2514546	-4.01285	6.00E-05	0.02238202
Apoe	4691.431124	1.067725892	0.2814384	3.793817	0.000148	0.02633044
Plekhf1	795.3515155	-1.13463267	0.3116494	-3.64073	0.000272	0.03752985
Fcgrt	1001.350564	1.35102035	0.3623378	3.728621	0.000193	0.02956953
Aen	663.2643529	-1.166717349	0.3115619	-3.74474	0.000181	0.02908386
Ap3s2	1406.915929	-1.034842912	0.2923134	-3.54018	0.0004	0.04636659
Hddc3	108.5802578	4.717597194	0.6502558	7.254986	4.02E-13	3.49E-09
Furin	96.02139208	2.197278133	0.5895621	3.726966	0.000194	0.02956953
Spns1	3955.180366	-1.086433946	0.2973064	-3.65426	0.000258	0.03677385
Sephs2	1891.923135	-1.066712904	0.2855396	-3.73578	0.000187	0.02956953
Cdc16	2927.126146	-1.112386851	0.2941001	-3.78234	0.000155	0.02649354
Gipc1	954.3440889	-1.178982867	0.2982832	-3.95256	7.73E-05	0.0228122
Gcdh	1756.44052	-1.133925189	0.3113155	-3.64237	0.00027	0.03752985
Ciapin1	1347.484573	-1.095702161	0.283728	-3.86181	0.000113	0.02370727
[illegible]230025D16Ri	2482.950648	-1.166320822	0.3008581	-3.87665	0.000106	0.02370727
Amotl2	561.3112888	-1.644531236	0.3522428	-4.66874	3.03E-06	0.00308356
Tex264	1932.093382	-1.083638681	0.2804057	-3.86454	0.000111	0.02370727
Plcd1	844.982273	-1.192141042	0.3221334	-3.70077	0.000215	0.0321377
Xylb	633.0534826	-1.261550002	0.3146544	-4.00932	6.09E-05	0.02238202
Zfp105	477.6885173	-1.9619835	0.3416256	-5.74308	9.30E-09	2.02E-05
Lars2	1284.161534	1.570551852	0.3356255	4.679477	2.88E-06	0.00308356
Mif	2622.846849	1.041075311	0.2739452	3.800305	0.000145	0.02633044
Chchd10	3473.28744	1.255166341	0.3272186	3.835865	0.000125	0.02473169
Cstb	11446.02272	1.052748079	0.2698742	3.900884	9.58E-05	0.02370727
Arid3a	135.9969871	2.096029555	0.5263385	3.982284	6.83E-05	0.02238202
Sgta	6985.651877	-1.025758145	0.2801864	-3.66099	0.000251	0.0364183
Cdk2	680.8597253	-1.52053962	0.3548184	-4.2854	1.82E-05	0.01057606
Ddx56	3481.348225	-1.094119865	0.261507	-4.1839	2.87E-05	0.01557561
Zkscan6	139.8144238	-1.718743332	0.4849638	-3.54407	0.000394	0.04636659
Txndc17	1428.479631	1.109062098	0.2944635	3.766382	0.000166	0.02770162
Slc46a1	791.0498265	-1.697803002	0.3644819	-4.65813	3.19E-06	0.00308356

Sdc1	266.502039	-1.627473405	0.454398	-3.5816	0.000341	0.0436758
Ifrd1	1095.66171	-1.43505665	0.2917217	-4.91927	8.69E-07	0.00150172
Cdca4	1465.670399	-1.147215621	0.2889376	-3.97046	7.17E-05	0.02238202
Bhmt2	1997.197901	-1.109676005	0.2726841	-4.06946	4.71E-05	0.02156988
Chchd1	1494.804352	1.055836755	0.2760619	3.824638	0.000131	0.02476077
Zfp623	271.1975653	-1.619436109	0.4023989	-4.02445	5.71E-05	0.02238202
Maf1	997.5041407	-1.152851877	0.2975726	-3.87419	0.000107	0.02370727
Gtse1	2435.368539	-1.084208828	0.2729904	-3.9716	7.14E-05	0.02238202
Ppp2r1a	3921.527343	-1.061512065	0.258188	-4.11139	3.93E-05	0.01952066
Rnps1	2599.556385	-1.2331517	0.2750197	-4.48387	7.33E-06	0.00579555
Dusp1	286.6401386	-1.570845609	0.4071949	-3.85772	0.000114	0.02370727
Med20	1884.053298	-1.030137442	0.278629	-3.69717	0.000218	0.0321377
Sh3gl1	1810.864023	-1.298807324	0.2974791	-4.36605	1.27E-05	0.00916922
Mta2	1031.69537	-1.126157061	0.3199995	-3.51925	0.000433	0.04915687
Scd2	2094.765053	1.083678201	0.2750622	3.939757	8.16E-05	0.0228122
Gata1	613.2215486	-1.60147818	0.3460238	-4.62823	3.69E-06	0.00320746
Ebp	1235.957039	1.179211032	0.2962784	3.980078	6.89E-05	0.02238202
Tbl1x	205.7998837	-1.764067462	0.440133	-4.00803	6.12E-05	0.02238202
Polr2m	2780.489579	-1.176648936	0.305018	-3.85764	0.000114	0.02370727
Cyb5a	1973.032826	1.556778079	0.269279	5.781284	7.41E-09	2.02E-05
Minos1	1277.981441	1.01928921	0.2856065	3.568858	3.59E-04	4.45E-02

Supplemental Table 6

metabolomic profiling of WT and Polg^{m} MII oocytes， related to Fig. 6

Compound	VIP	Polg^{m} vs. WT Fold change	P value
ignificantly changed metabolites			
S-Adenosylmethionine	1.34	0.46	0.04
Homocysteine	1.36	0.64	0.04
Taurine	1.37	3.71	0.01
Oxidized glutathione	1.26	1.46	0.09
Oxoglutaric acid	1.23	0.54	0.13
Succinic acid	1.39	0.38	0.01
L-Glutamine	1.27	4.28	0.04
1-Palmitoyl-sn-glycero-3-ph	1.39	12.67	0.01
2-Hydroxycaproic acid	1.12	0.09	0.08
3,4,5-Trimethoxycinnamic aci	1.36	0.32	0.04
4-Aminohippuric acid	1.38	0.68	0.01
5'-Methylthioadenosine	1.29	2.17	0.08
Adenosine	1.23	0.73	0.12
Aminocaproic acid	1.37	0.52	0.02
Androstenedione	1.40	1.79	0.00
Benzenebutanoic acid	1.40	1.92	0.00
Capric acid	1.28	0.30	0.09
Cortisone	1.17	2.55	0.12
Dihydroxyacetone phosphate	1.18	3.11	0.09
Ethylmalonic acid	1.17	1.57	0.14
L-Cystine	1.30	2.45	0.11
L-Proline	1.34	0.76	0.06
Pantothenol	1.25	0.43	0.16
Spermine	1.32	0.40	0.08
trans-Ferulic acid	1.23	0.70	0.15
Tris(hydroxymethyl)aminome	1.27	1.57	0.08
Xanthosine	1.30	0.47	0.11
Lactic acid	1.25	0.28	0.10
Pyruvic acid	1.25	1.67	0.14
FAD	1.38	0.75	0.02
Unchanged metabolites			
17-Hydroxyprogesterone	0.98	5.00	0.24
Deoxycytidine	0.96	0.12	0.42
2-Hydroxypalmitic Acid	1.03	2.84	0.22
2-Indolecarboxylic acid	0.70	0.99	0.85
3-Hydroxyphenylacetic acid	0.91	2.81	0.42
3-Indoleacetonitrile	0.87	0.72	0.72
3-Methyladenine	0.97	0.70	0.42
3-Methylindole	0.97	0.80	0.42
3-Phenylbutyric Acid	1.16	1.29	0.24
3-Pyridylacetic acid	0.54	1.16	0.46
Ureidopropionic acid	0.92	4.79	0.42
4-Methoxyphenylacetic acid	0.91	1.09	0.38
4-Methylcatechol	0.80	0.62	0.43
5-Hydroxymethyl-2-Deoxyur	1.03	0.49	0.44
7b-Hydroxycholesterol	0.81	0.62	0.50
L-Acetylcarnitine	0.72	0.95	0.45
Adenine	0.82	0.99	0.62
3'-AMP	0.89	1.09	0.22
Agmatine Sulfate	0.97	0.59	0.42
Allantoin	0.90	1.36	0.55
Argininosuccinic acid	1.12	0.88	0.33
Ascorbic acid	0.45	1.57	0.69

Carnosine	0.91	1.66	0.42
Cholesterol	0.09	1.08	0.76
Cholic acid	0.91	1.80	0.42
cis-Aconitic acid	0.89	1.34	0.51
Citraconic acid	0.91	1.05	0.19
Cortisol	0.97	0.88	0.42
Cuminaldehyde	0.96	0.98	0.42
Cyclic AMP	1.09	1.64	0.24
Cytidine	0.96	0.40	0.42
Cytosine	0.96	0.34	0.42
5-Aminolevulinic acid	1.15	0.77	0.30
Deoxyinosine	0.91	1.28	0.42
D-Glutamic acid	0.68	1.12	0.32
Dimethylbenzimidazole	0.91	1.31	0.35
Glyceraldehyde	0.99	0.23	0.31
Isocitric acid	0.81	0.89	0.64
Mannose 6-phosphate	0.97	0.69	0.42
Dodecanedioic acid	0.86	0.80	0.40
Dodecanoic acid	1.23	0.84	0.13
Neopterin	0.97	0.84	0.42
D-Glucaric acid	0.87	0.88	0.72
Estriol	0.91	1.15	0.19
Gluconolactone	0.66	0.96	0.80
Glucose 6-phosphate	1.10	1.72	0.20
Glutaconic acid	0.88	1.38	0.44
Glycerophosphocholine	0.81	0.77	0.54
Glycine	0.68	1.02	0.74
Glycocholic acid	0.92	1.36	0.37
Glycolic acid	1.10	1.68	0.33
Guanidine	0.97	0.42	0.46
Guanine	0.90	1.13	0.25
Hexadecanedioic acid	0.79	1.03	0.74
Homoveratric acid	0.24	1.09	0.65
Hydroxyurea	0.37	1.06	0.64
Hyodeoxycholic acid	0.91	37.76	0.42
Hypotaurine	0.87	1.10	0.28
Indoleacrylic acid	0.91	1.82	0.42
Inosinic acid	0.01	1.00	0.86
Inosine	0.99	1.54	0.38
Itaconic acid	0.91	1.93	0.42
L-Aspartyl-L-phenylalanine	0.81	1.05	0.76
L-Carnitine	1.38	0.79	0.02
L-Dopa	0.97	0.96	0.35
L-Histidine	0.97	0.48	0.42
L-Leucine	0.82	0.95	0.90
L-Lysine	0.89	1.26	0.52
Norvaline	0.88	1.06	0.21
L-Serine	0.88	0.70	0.58
Thyroxine	0.85	0.42	0.55
L-Tryptophan	0.81	0.93	0.88
Maleic acid	0.91	17.33	0.42
(-)-Matairesinol	0.97	0.53	0.42
N-Acetylglutamic acid	0.97	0.60	0.41
N-Acetyl-L-methionine	1.11	0.77	0.24
N-Acetyl-L-phenylalanine	0.99	0.88	0.37
N-Acetyl-L-tyrosine	0.91	2.21	0.42
N-Acetylneuraminic acid	0.96	0.33	0.42
N-Acetylvaline	0.96	0.69	0.42

N-Formyl-L-methionine	0.91	1.10	0.10
Niacinamide	0.73	0.98	0.53
N-Oleoylethanolamine	0.91	2.40	0.42
Octadecanamide	0.44	0.74	0.66
Pyridoxal	0.91	3.67	0.42
Pyridoxal 5'-phosphate	0.78	2.11	0.50
Pyroglutamic acid	1.03	0.48	0.43
Quinic acid	1.16	0.79	0.27
Ranitidine	0.90	0.70	0.61
Retinal	0.97	0.99	0.25
Rhamnose	0.85	0.67	0.75
Riboflavin	1.01	0.69	0.30
Serotonin	0.96	0.76	0.42
Sorbitol	0.82	0.59	0.46
Tetradecanedioic acid	0.89	0.82	0.65
Thiamine	0.99	0.71	0.38
Thymidine	0.91	1.54	0.41
Farnesol	0.91	1.25	0.42
Tryptamine	0.83	0.72	0.63
Uridine	0.82	0.85	0.75
Valeric acid	0.91	1.48	0.40
NAD	0.91	0.93	0.49
Glucose	0.99	0.23	0.31
Citric acid	1.10	1.39	0.23
Glutathione	0.89	1.03	0.16
Malic acid	1.08	0.74	0.35
Acetoacetic acid	1.07	0.88	0.30
L-Cystathionine	0.78	0.87	0.86
5-Methyltetrahydrofolic acid	1.00	1.76	0.30
Uridine diphosphate-N-acety	0.96	0.63	0.42
Fumaric acid	0.91	1.40	0.43
Methionine	0.60	0.99	0.64
O-Phosphoethanolamine	1.02	1.40	0.32
