# Supplementary material for: Increased mtDNA mutation frequency in oocytes causes epigenetic alterations and embryonic defects
Source: Natl Sci Rev. 2022 Jul 13;9(10):nwac136. doi: 10.1093/nsr/nwac136 (PMC9616472; doi:10.1093/nsr/nwac136)
Supplement: nwac136_Supplemental_files [file nwac136_supplemental_files.zip › Supplemental_figures_and_legends.pdf]

Supplemental Figures

Figure S1

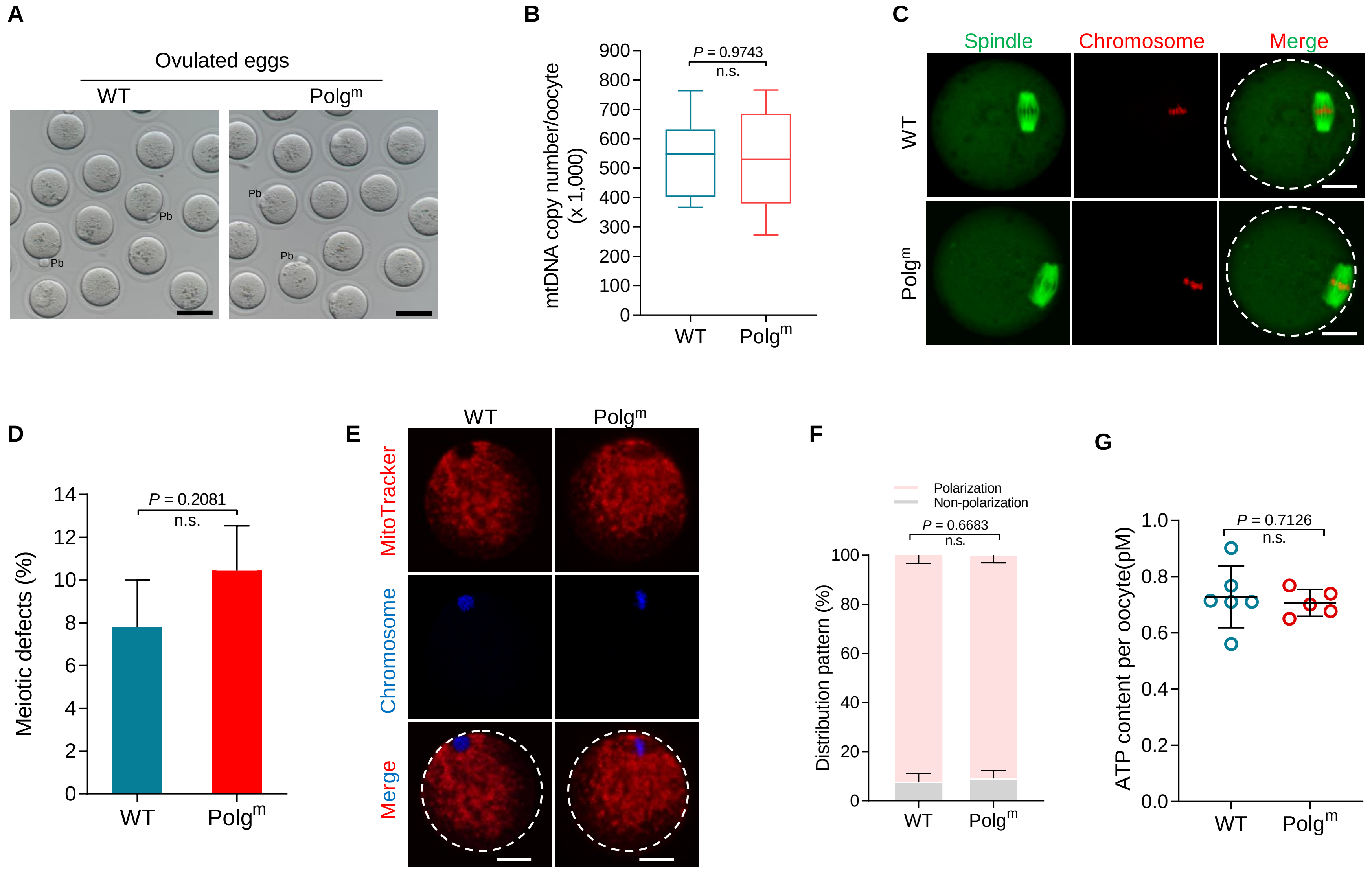

Figure S2

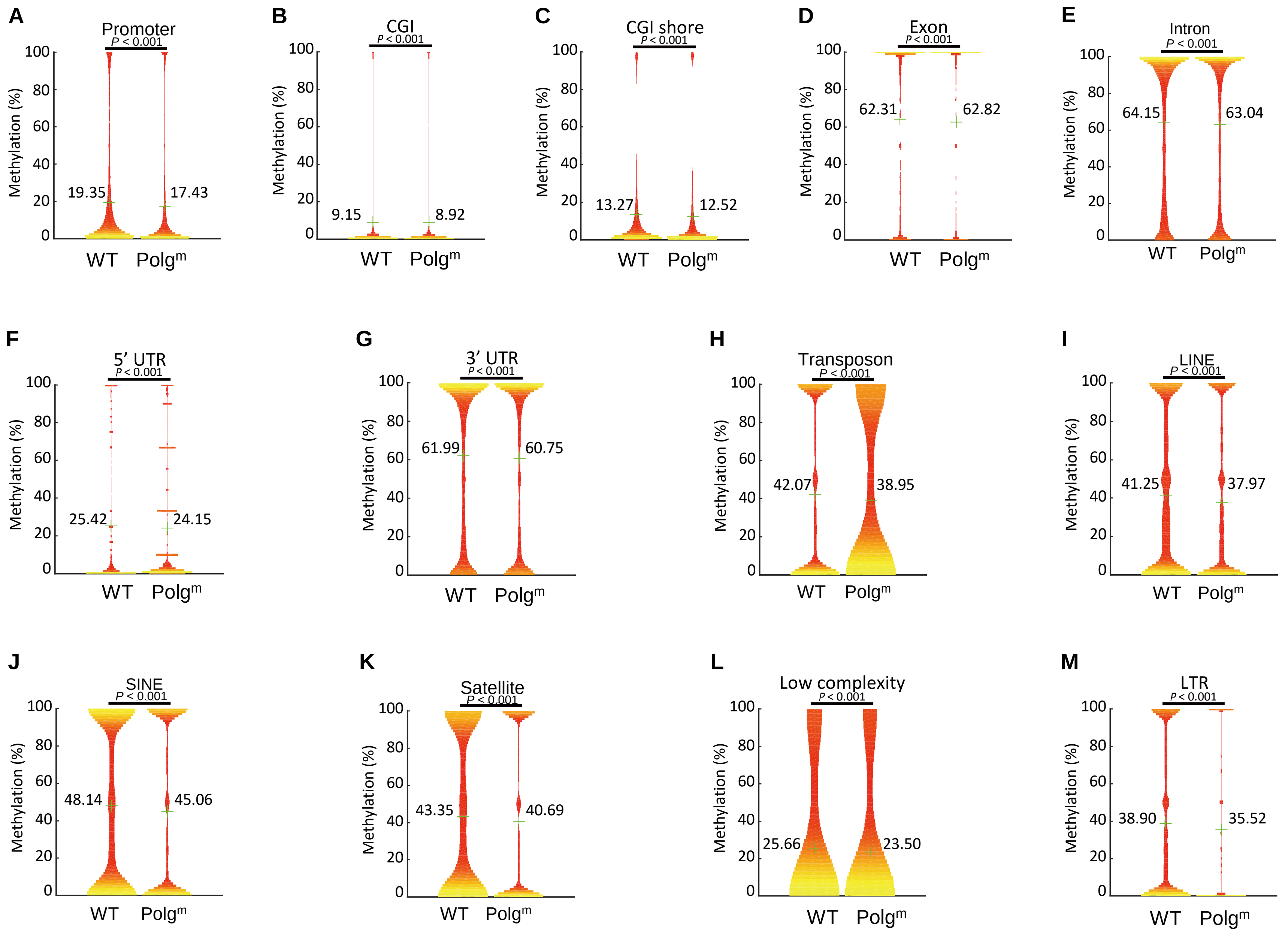

A
Promoter
P < 0.001
Methylation (%)
19.35
17.43
WT
Polgm
B
CGI
P < 0.001
9.15
8.92
C
CGI shore
P < 0.001
13.27
12.52
D
Exon
P < 0.001
62.31
62.82
E
Intron
P < 0.001
64.15
63.04
F
5' UTR
P < 0.001
25.42
24.15
G
3' UTR
P < 0.001
61.99
60.75
H
Transposon
P < 0.001
42.07
38.95
I
LINE
P < 0.001
41.25
37.97
J
SINE
P < 0.001
48.14
45.06
K
Satellite
P < 0.001
43.35
40.69
L
Low complexity
P < 0.001
25.66
23.50
M
LTR
P < 0.001
38.90
35.52

Figure S3

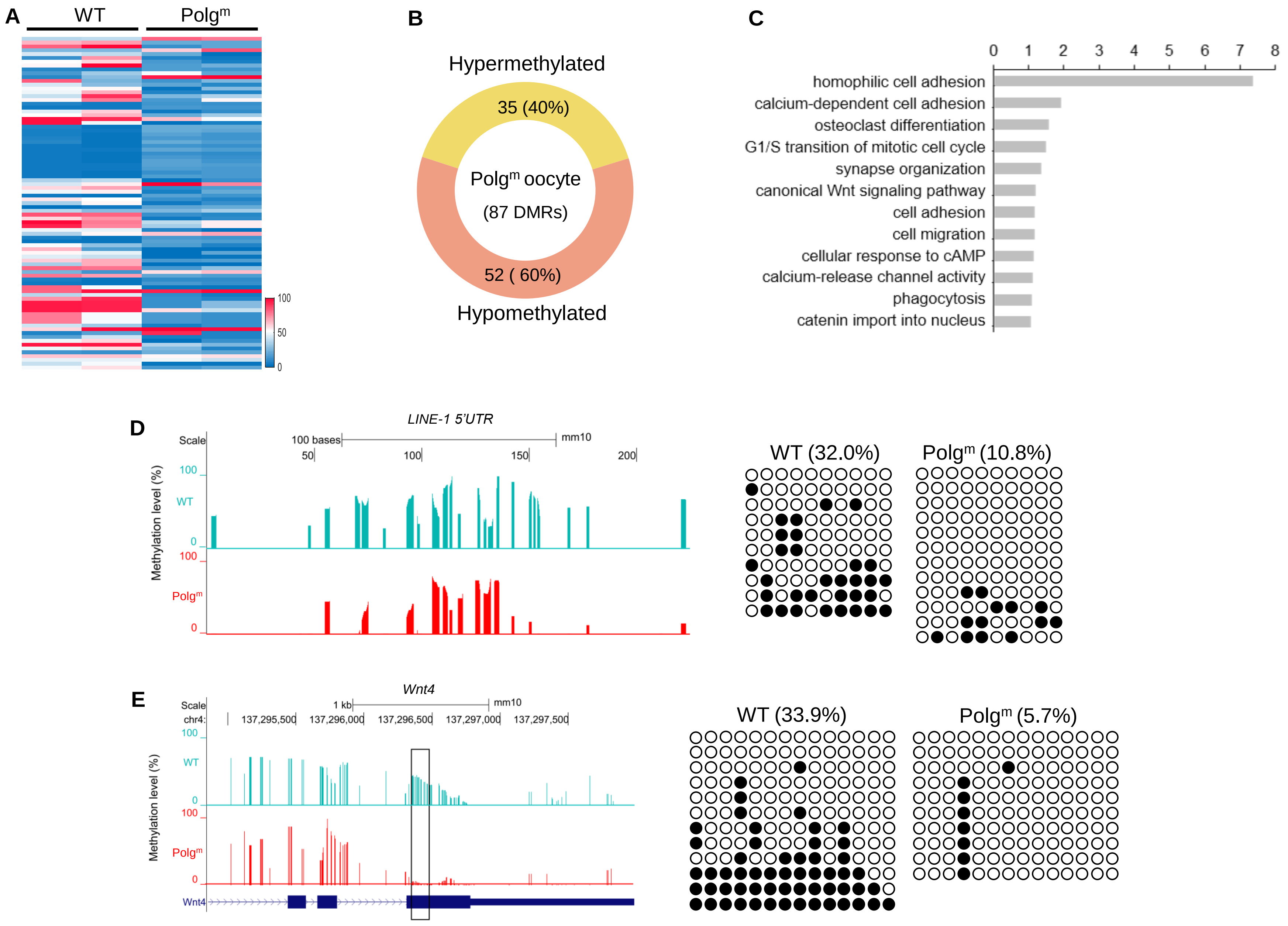
A
WT
Polgm
100
50
0
B
Hypermethylated
35 (40%)
Polgm oocyte
(87 DMRs)
52 (60%)
Hypomethylated
C
0 1 2 3 4 5 6 7 8
homophilic cell adhesion
calcium-dependent cell adhesion
osteoclast differentiation
G1/S transition of mitotic cell cycle
synapse organization
canonical Wnt signaling pathway
cell adhesion
cell migration
cellular response to cAMP
calcium-release channel activity
phagocytosis
catenin import into nucleus
D
LINE-1 5'UTR
Scale
100 bases
mm10
50
100
150
200
Methylation level (%)
100
WT
0
100
Polgm
0
WT (32.0%)
Polgm (10.8%)
E
Wnt4
Scale
1 kb
mm10
chr4:
137,295,500
137,296,000
137,296,500
137,297,000
137,297,500
100
WT
0
100
Polgm
0
Wnt4
WT (33.9%)
Polgm (5.7%)

Figure S4

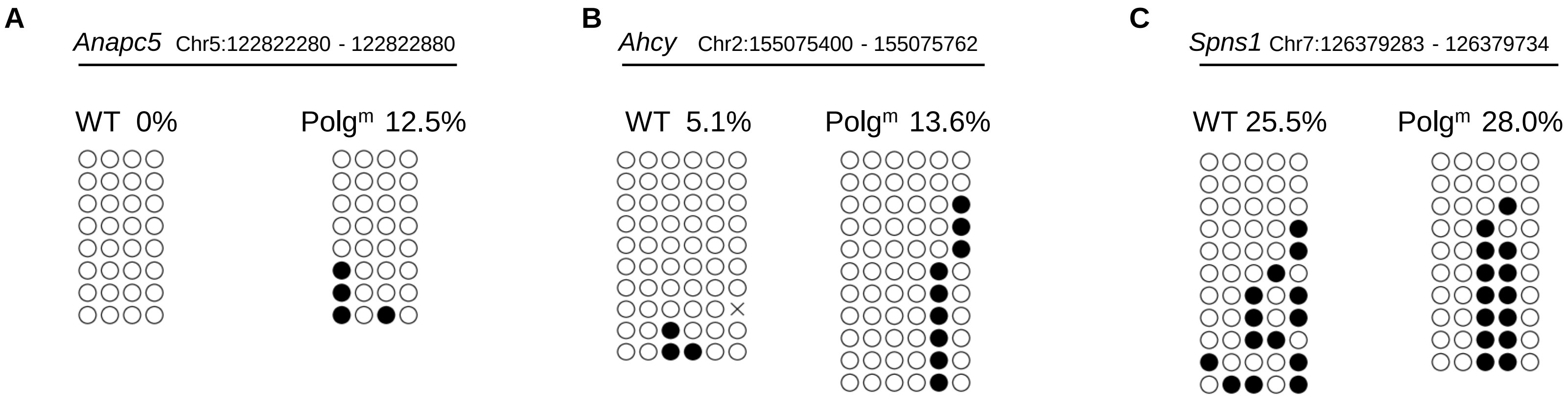

A
Anapc5 Chr5:122822280 - 122822880
WT 0%
Polg^m 12.5%
B
Ahcy Chr2:155075400 - 155075762
WT 5.1%
Polg^m 13.6%
C
Spns1 Chr7:126379283 - 126379734
WT 25.5%
Polg^m 28.0%

Figure S5

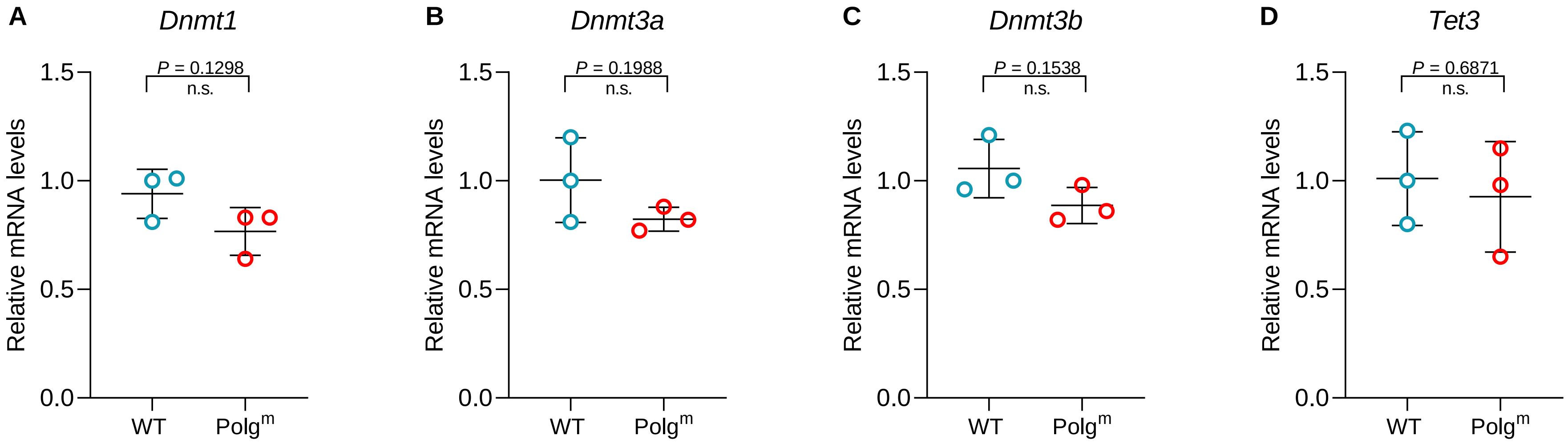
A
Dnmt1
P = 0.1298
n.s.
B
Dnmt3a
P = 0.1988
n.s.
C
Dnmt3b
P = 0.1538
n.s.
D
Tet3
P = 0.6871
n.s.
Relative mRNA levels
1.5
1.0
0.5
0.0
WT
Polgm

Figure S6

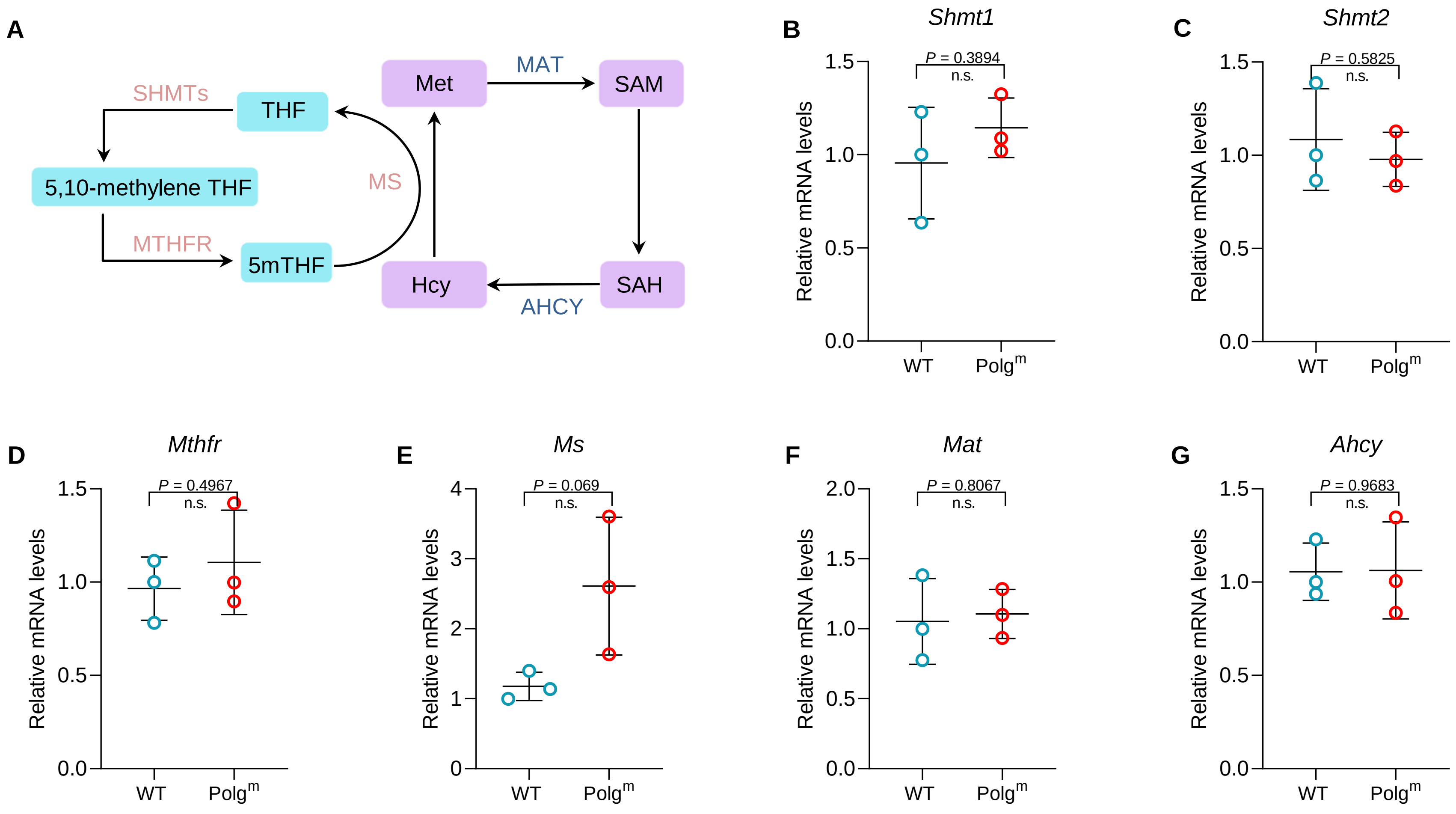

THF: Tetrahydrofolate; 5,10-methylene THF: 5,10-methylene tetrahydrofolate; 5mTHF: 5-methyl-tetrahydrofolate; Met: methionine; SAM: S-adenosylmethionine; SAH: S-adenosylhomocysteine; Hcy: homocysteine *Shmt*: serine hydroxymethyltransferase; *Mthfr*: methylenetetrahydrofolate reductase; *Ms*: Methionine synthase; *Mat*: Methionine adenosyltransferase 1a; *Ahcy*: S-adenosylhomocysteine hydrolase

Figure S7

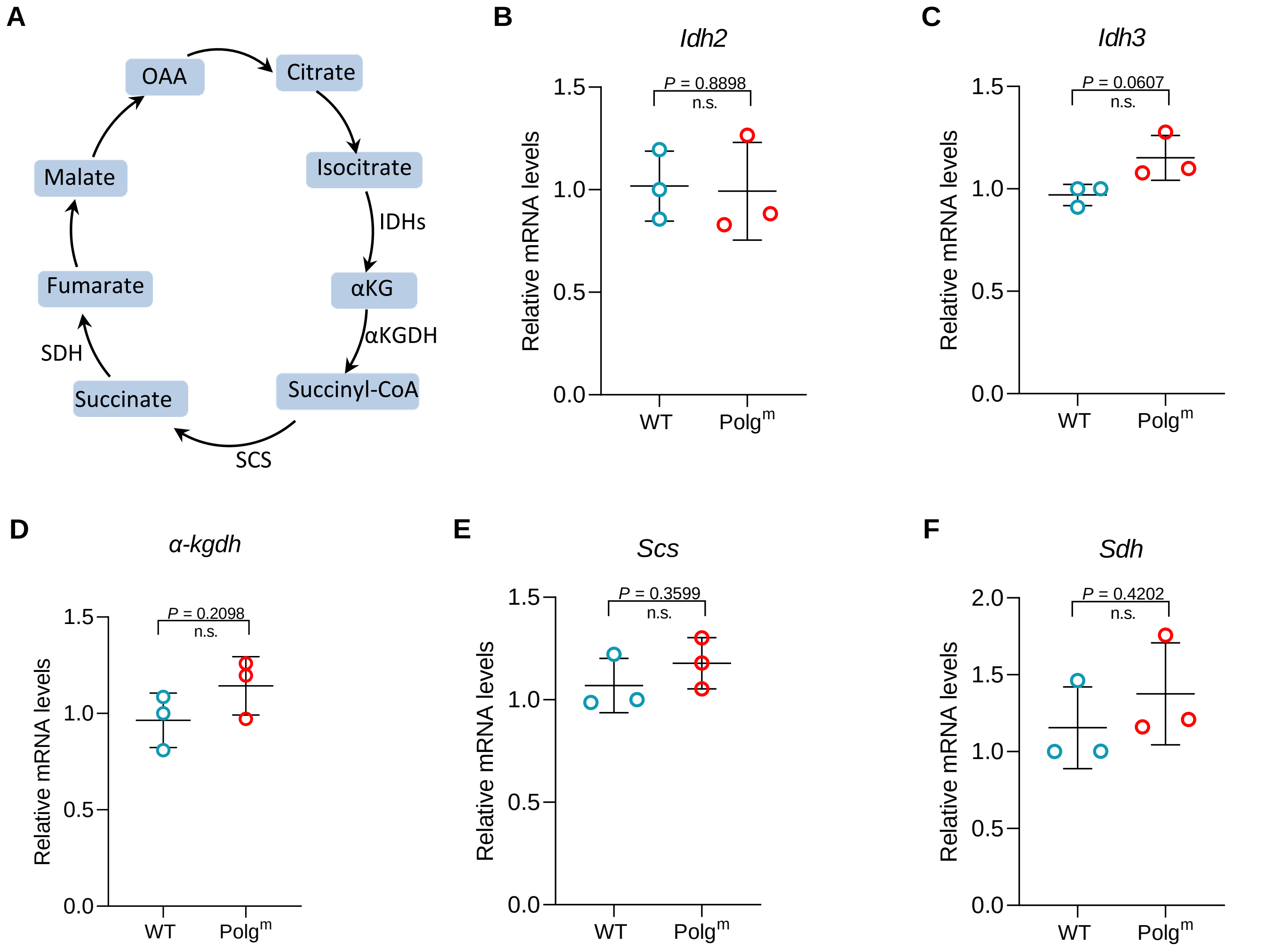

Idh2: isocitrate dehydrogenase 2; *Idh3*: isocitrate dehydrogenase 3 alpha; *α-kgdh*: α-ketoglutarate dehydrogenase; *Scs*: succinyl-CoA synthetase; *Sdh*: succinate dehydrogenase complex, subunit A

Figure S8

A

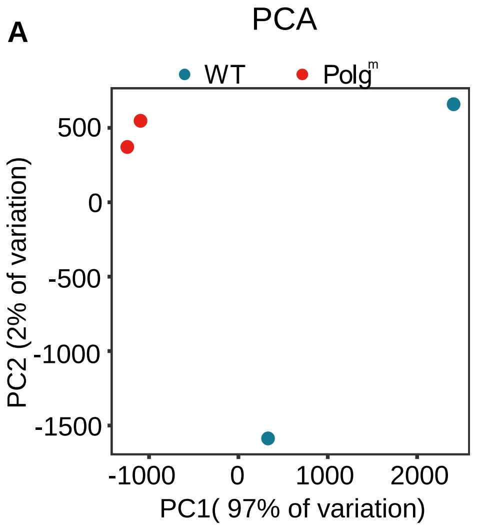

B

Sample		Mapping efficiency (%)	Average depth per strand	CpG coverage (%)	Mean CG methylation (%)	Correlation coefficient of CG methylation (20-kbp windows)
WT oocyte	rep 1	58.00%	2.14	85	41.46	0.97
	rep 2	56.90%	2.11	85	38.86	
Polg^m oocyte	rep 1	54.60%	2.01	81	35.87	0.9718
	rep 2	54.60%	1.88	83	36.07	

Figure S9

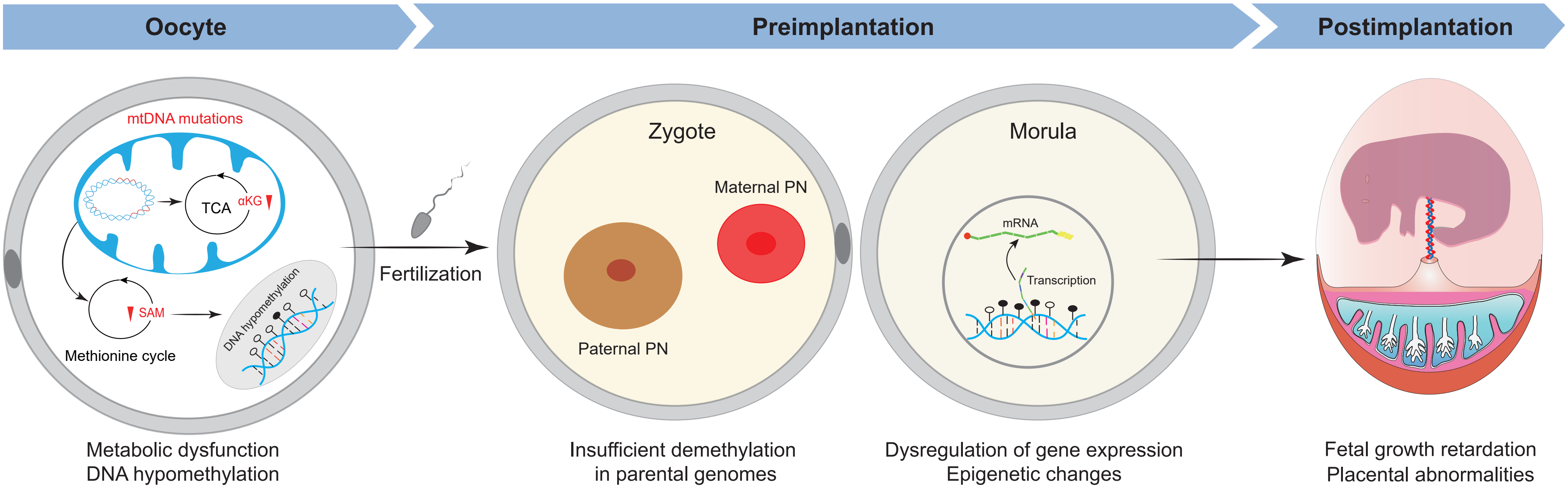
Oocyte
Preimplantation
Postimplantation
mtDNA mutations
TCA
αKG
SAM
Methionine cycle
DNA hypomethylation
Fertilization
Zygote
Maternal PN
Paternal PN
Morula
mRNA
Transcription
Metabolic dysfunction
DNA hypomethylation
Insufficient demethylation
in parental genomes
Dysregulation of gene expression
Epigenetic changes
Fetal growth retardation
Placental abnormalities

Supplemental Figure Legends

Fig. S1 Evaluation of oocyte quality from Polg^m mice. (A) Representative bright-field images of MII oocytes retrieved from WT and Polg^m mice. Scale bar, 50 µm. (B) Quantitative analysis of mtDNA copy number in oocytes from WT and Polg^m mice (n=15 for each group). (C) Oocytes from WT and Polg^m mice were immunolabeled with α-tubulin to labeled spindle (green) and co-stained propodium iodide to visualize chromosomes (red). Dash line indicates the oocyte border. (D) Quantification of WT (n=89) and Polg^m oocytes (n=92) with spindle defects or chromosome misalignment. Scale bar, 20 µm. (E) Distribution of mitochondria in WT and Polg^m oocytes. MII oocytes were labeled with MitoTracker red to visualize mitochondria (red), and co-stained with Hoechst 33342 for chromosomes (blue). Scale bar, 20 µm. (F) Classification of oocyte mitochondrial distribution. Oocytes with highly polarized mitochondria were classified as 'polarization'; oocytes with other distribution patter were categorized as 'non-polarization' (WT, n = 56; Polg^m, n = 63). (G) Intracellular ATP contents of oocytes from WT and Polg^m mice. MII oocytes were obtained from 19-21-week old mice for all experiments. Data are expressed as mean ± SD from three independent experiments. Student's *t* test (two-tailed) was used for statistical analysis; n.s., not significant.

Fig. S2 Global DNA hypomethylation across genomic features in Polg^m oocytes. (A-M) Violin plots showing the methylation levels of the promoter (A), CGIs (B), CGI shore (C), exon (D), intron (E), UTR (F-G), as well as the repetitive elements (H-M) in oocytes from WT and Polg^m mice. Mean methylation levels are marked by the numerical value and green cross. Statistical analyses were performed with a bootstrap test.

Fig. S3 Analysis of differentially methylated regions (DMRs) between WT and Polg^m oocytes. (A) Heatmap of 87 DMRs, with each line representing a DMR identified between WT and Polg^m oocytes. (B) Doughnut chart illustrating the total number of DMRs identified between WT and Polg^m oocytes, and the proportion of hyper- and hypo-DMRs. (C) Gene ontology (GO) analysis for the DMRs-associated genes. GO terms with Benjamin score < 0.05 are shown in the graph. (D-E) Left, graphical representation of the methylation pattern at test locus LINE-1 and Wnt4 in WT and Polg^m oocytes. The region marked by gray box was chosen for further validation. Right, bisulfite

sequencing for verifying the methylation state of LINE-1 5′-UTR and Wnt4 in WT and Polg^{m} oocytes. Open and solid circles represent unmethylated and methylated CpGs, respectively. LINE-1, 10 CpG sites; Wnt4, 14 CpG sites.

Fig. S4 Validation of the methylation status of three genes in Polg^{m} morula. BS-Seq analysis examining the methylation status of *Anapc5* (A), *Ahcy* (B) and *Spns1* (C) promoter in WT and Polg^{m} morula. Circles represent CpG dinucleotides either unmethylated (open) or methylated (closed). The gene promoter positions selected for validation were indicated above.

Fig. S5 Expression analysis of DNA methyltransferase and demethylase in WT and Polg^{m} oocytes. Relative mRNA levels of *Dnmt1*, *Dnmt3a, Dnmt3b* and *Tet3* are determined by real-time RT-PCR. Error bars, SD. Student's *t* test was used for statistical analysis. n.s., not significant.

Fig. S6 qRT-PCR analysis verifying the expression of genes involved in one-carbon metabolism pathway. (A) Schematic diagram of one-carbon metabolism pathway. (B-G) Relative abundance of the indicated transcripts in WT and Polg^{m} oocytes. Error bars, SD. Student's *t* test was used for statistical analysis. n.s., not significant.

Fig. S7 qRT-PCR analysis verifying the expression of genes involved in TCA cycle. (A) Schematic diagram of TCA cycle pathway. (B-F) Relative abundance of the indicated transcripts in WT and Polg^{m} oocytes. Error bars, SD. Student's *t* test was used for statistical analysis. n.s., not significant.

Fig. S8 Summary of BS-Seq data quality. (A) Principal Component Analysis plot. Polg^{m} oocytes cluster separately from WT oocytes in PC1. (B) Sequencing and mapping summary of BS-Seq data. Two samples for each group were used for global DNA methylation analysis.

Fig. S9 Diagram illustrating the proposed mechanisms for epigenetic changes and embryonic defects induced by oocyte mtDNA mutations. Increase of mtDNA mutation frequency in oocytes results in metabolic dysfunction. On one hand, the diminished methionine cycle activity and insufficient provision of methyl donors SAM in Polg^{m} oocytes may contribute to the global loss of DNA methylation. On the other hand, the downregulated TCA cycle, specifically αKG reduction, disrupts the epigenetic

reprogramming in zygotes and the subsequent embryonic gene expression, which is, at least in part, responsible for the developmental abnormalities of postimplantation embryos derived from $Polg^{m}$ oocytes.
