# Supplementary material for: Increased mtDNA mutation frequency in oocytes causes epigenetic alterations and embryonic defects
Source: Natl Sci Rev. 2022 Jul 13;9(10):nwac136. doi: 10.1093/nsr/nwac136 (PMC9616472; doi:10.1093/nsr/nwac136)
Supplement: nwac136_Supplemental_files [file nwac136_supplemental_files.zip › Supplemental_Materials_and_Methods.docx]

**MATERIALS AND METHODS**

**Mice.** All animal experiments follow the rules and guidelines of the local animal ethical committee and the Animal Care and Use Committee of Nanjing Medical University. Heterozygotes for the mitochondrial polymerase gamma knock-in mutation (C57BL/6J, PolgA^wt/mut^) were purchased from The Jackson Laboratory (Stock NO: 017341). Generation of Polg^mut^ transgenic mice has been previously reported (1). Briefly, an AC-->CT two-base substitution was introduced to mouse DNA polymerase γ (Polg) sequence, corresponding to the positions 1054-1055 in exon 3. The point mutations create a D257A amino acid substitution in the conserved N-terminal exonuclease domain II of PolgA, impairing its proofreading ability. Germline transmission of the mutation produces Polg^wt/mut^ mice, which were intercrossed to generate homozygous Polg^mut/mut^ mice (Polg^m^ mice) and Polg^wt/wt^mice (WT mice).

To test the fecundity, five individually housed WT and Polg^m^ mice were mated with a stud male mouse validated to be fertile at the age of 8 weeks. Cages were examined twice a week and the number of litter and litter size was recorded up to 6 months. To induce superovulation, female mice at indicated time points were intraperitoneally injected with 5IU of pregnant mare’s serum gonadotropin (PMSG), followed by 5 IU human chorionic gonadotropin (hCG) after 44 h. The oviducts were dissected and cumulus-oocyte complexes were collected at 14 h of hCG administration. After treatment with 0.5 mg/ml hyaluronidase (Sigma, St. Louis, MO, USA) in M16 medium, oocytes were collected for subsequent experiments.

***In vitro* fertilization and embryo evaluation.** IVF procedures were performed as we described previously (2). In brief, normal sperm were collected from the cauda epididymides of ICR mice aged 12-20 weeks, and left to capacitate for 1 h in HTF medium (Millipore, Merck) supplemented with 10mg/mL BSA. Intact cumulus-oocyte complexes were mixed with 1~2 x 10^6^ sperm/mL in HTF drops. For αKG supplement experiment, MII oocytes were collected and cultured in HTF medium containing 150 μM αKG before and after fertilization, αKG concentration was selected based on the published report (3). After co-incubation for 6~9 h, zygotes were washed to removed cumulus cells and excess sperm, and then transferred into KSOM medium (Millipore, Merck) for further culture. Early embryo development potential was assessed at the indicated time points during culture. For the embryo transfer experiment, 2-cell stage embryos were transferred into the oviducts of ICR pseudopregnant surrogate mothers at a ratio of 15 embryos per mouse. To assess the fetal development in uteri, surrogate mothers were sacrificed on day 18.5, fetuses and placentas were isolated for analysis.

**Immunofluorescence.** Oocytes were fixed in 4% paraformaldehyde (PFA) for 30 min at room temperature, and permeabilized with 0.5% Triton X-100 for 20 min. After being blocked with 1% BSA in PBS, oocytes were incubated with FITC-conjugated anti–α-tubulin antibody (Sigma-Aldrich, F2168). Chromosome was stained with propidium iodide (red) for 10 min. Following three washes with PBS, samples were mounted on glass slides in a drop of anti-fade medium (Vectashield, CA, USA), and then examined with a Laser Scanning Confocal Microscope (LSM 710; Zeiss, Oberkochen, Germany).

5mC/5hmC staining of zygotes was carried out according to our protocol (2). Briefly, permeabilization DNA was denatured in 4 M HCl for 10 min at room temperature, followed by neutralized in Tris-HCl pH 8.0 for 10 min. Samples were then blocked overnight at 4 °C before incubation with the primary antibodies. Following extensive wash in PBST, samples were incubated with secondary antibodies for 4 h at room temperature. After three washes with PBST, samples were mounted on glass slides. Fluorescence intensity was quantified using ImageJ software as we described previously (2). Paternal and maternal pronuclei were determined based on their size and the distance to polar body, as described previously (4).

**mtDNA mutation detection.** About 10~20 oocytes were harvested in a PCR tube with 10 μl lysis buffer (containing 50 mM Tris-HCl, 0.1 mM EDTA, 0.5% Tween-20, and 200 μg/mL proteinase K), and incubated at 55 ^o^C for 2 h. Proteinase K was inactivated at 95 ^o^C for 10 min, and then samples were processed as previously described (1). In brief, a mtDNA fragment of 525 bp spanning part of the *Cytb* (cytochrome b) gene and the D-loop control region and mtDNA fragment of 487 bp within the *Cox1* (cytochrome c oxidase) gene were amplified using primers listed in Supplemental Table 1. PCR products were cloned into the pMD19-T vector with TA Cloning Kit (TaKaRa, 6013). 100 positive colonies of each sample were selected for sequencing using standard M13-47 primers. DNA sequences were aligned to the B6 mtDNA reference sequence (Accession: NC 005089) via online Blast method at NCBI website.

**Quantification of mtDNA copy number.** mtDNA extraction and quantitative reverse transcription PCR procedure have been described previously (5). In brief, a single oocyte was loaded in a PCR tube containing 10 μl lysis buffer, and was incubated at 55 ^o^C for 2 h, then 95 ^o^C for 10 min for inactivation of Proteinase K. The samples were used for real-time PCR by employing ABI system and mouse mtDNA-specific primers (Supplemental Table 1). To obtain the standard curve, PCR products amplified with B6 forward and B6 reverse primers were ligated into T-vector. Five 10-fold serial dilutions of purified plasmid standard DNA were used to generate the standard curve. All measurements were performed in triplicate.

**ROS evaluation.** To detect the ROS production in living oocytes, oocytes were incubated in HEPES medium with 5 μmol/L CM-H_2_DCFDA (Life Technologies, Invitrogen TM, Ca#: C6827) for 30 min at 37 ^o^C in 5% CO_2_ incubator. Following several washes, 10~15 oocytes were transferred to a live cell-imaging dish, and images were taken using a Zeiss Laser Scanning Confocal Microscope (LSM 710; Zeiss, Oberkochen, Germany). The mean fluorescence intensity of cells detected at 488 nm excitation was determined.

**Detection of total ATP content**

Total ATP content in pools of 20 oocytes was determined by using the bioluminescent somatic cell assay Kit (Sigma), following the procedure as described(6). Standard curve containing six-point (0, 0.1 0.5, 1.0, 10, and 50pmol of ATP) was generated in each experiment and the ATP content was calculated by using the formula derived from the linear regression of the standard curve.

**Analysis of mitochondrial membrane potential**

Mitochondrial membrane potential in oocytes was determined using JC-1 (5,5’,6,6’-

tetrachloro-1,1’,3,3’-tetraethylbenzimidazolylcarbocyanine iodide; Invitrogen), a dye that forms J-aggregates (red fluorescence) when the mitochondrion has a high ΔΨm, and remains in monomers (green fluorescence) if ΔΨm is low. Oocytes were incubated in M2 medium containing JC-1 (10μg/mL) at 37°C for 15 min. Following incubation, samples were immediately observed using a confocal laser microscope (LSM 710; Zeiss, Oberkochen, Germany). The captured images were processed using ImageJ software. Mitochondrial membrane potential was assessed by measuring the red:green fluorescence ratio. The average intensity of every oocyte was used for statistical analysis.

**Evaluation of mitochondrial distribution.** To examine mitochondrial distribution in living oocytes, MII oocytes were incubated in HEPES medium with 0.5 μmol/L Mito-Tracker red (Life Technologies, Invitrogen TM, Ca#: M22425) and Hoechst 33342 for 30 min at 37 ^o^C in 5% CO_2_ incubator. Following three washes, 10~15 oocytes were transferred to a live cell-imaging dish, and images were taken using a Zeiss Laser Scanning Confocal Microscope.

**Quantitative reverse transcription PCR**. Total RNA was extracted from 50 oocytes using Arcturus PicoPure RNA Isolation Kit (Applied Biosystems, CA, USA). Isolated RNA was converted into cDNA using a cDNA Synthesis Kit (Qiagen, Germany). Real-time PCR was performed with SYBR Green using an ABI StepOnePlus Real-time PCR system (Applied Biosystems, CA, USA). Glyceraldehydes-3-phosphate dehydrogenase (*Gapdh*) was used as an internal control. Experiments were performed in triplicate. Values are expressed relative to control values, as appropriate, set as 1. Primers used for qPCR can be found in Supplemental Table 1.

**Spindle transfer** Female mice were superovulated using serial injection of PMSG and hCG. MII oocytes were collected after 14 h hCG administration. For spindle transfer, the spindle-chromosome complex of an individual oocyte from each group was gently aspirated into a pipette using a Piezo-drill micromanipulator in M2 medium supplemented with 5μg/ml cytochalasin B (Sigma). Next, the spindle-chromosome complex was transferred to the hemagglutinating virus of Japan envelope (Cosmo Bio) drop and then gently injected into the perivitelline space of an enucleated recipient oocyte. The reconstructed oocytes were retained in the manipulation drop for 20 min to complete the fusion, and then were used for in vitro fertilization.

**Oocyte methylome profiling.** The DNA methylome libraries were constructed as described previously (7). Briefly, 20 denuded MII oocytes collected from 5 mice were lysed in buffer containing 0.5 µl proteinase K for 1 h at 37°C. DNA was converted by the EZ DNA Methylation-Gold kit (Zymo Research) according to the instructions. The converted DNA was subjected to library construction, and the quality and quantity of the purified library were assessed using Agilent Bioanalyzer and StepOnePlus Real-Time PCR System (Applied Biosystems). Libraries were prepared for 125-bp paired-end sequencing on an Illumina HiSeq 2500.

**Analysis of methylome data.** Trim Galore was used to trim the poor-quality bases and adapter sequences from raw sequence with auto-detected parameters. Cleaned data were mapped to mm10 reference genome using Bismark (8) with following parameters: ‘single-end, options –non-directional,-bam, —bowtie2’. Bam files obtained from read1 and read2 were merged and processed by Bismark methylation extractor with paramters ‘--bedGraph’. To calculate the mean methylation level of variable genomic features, bedmap was applied with default parameter ‘--faster’ on the CpG methylation files. Promoters, exons, introns, UTR’3 and UTR’5 were obtained from UCSC RefSeq (9). Here the promoters were defined as the 2kb upstream from TSSs (transcriptional start sites). Repeat elements were extracted from the pre-defined dataset from Repeatmasker (10). CpG islands (CGIs) were extracted from the CpG islands track of the UCSC Genome Browser, and CGI shores were defined as the regions 2kb around from the CGI.

To identify the differential methylated regions (DMRs) between groups, metilene was used with default parameter in mode 1 (11). Differential methylated promoters (DMPs) were also identified by metilene with parameter in mode 2. Significant DMRs and DMPs were defined as DMRs with q value < 0.05 and |methylation difference| > 0.1. Gene ontology (GO) enrichment analysis was performed on the set of genes which were closest to DMRs using DAVID (12). GO results were filtered with FDR < 0.05. Methylation level profile on specific genomic region is drawn by IGV. To profile the methylation level across the potential LINE UTR’5 region on the whole genome, we mapped the sequence of LINE UTR’5 to the mm10 genome with STAR with ‘--two pass Mode Basic --out SAM type BAM Unsorted’ and retained all the possible mapping results. Then we extracted the mapped regions and calculated the mean methylation level on each base of the three repeats in each two groups. Hypomethylated/hypermethylated domains, and gICR regions were obtained from previously published reports (13, 14). In addition, information on BS-Seq data quality is summarized in Fig. S8.

**Bisulfite pyrosequencing.** The methylation profile of 2 DMR regions (LINE-1 and Wnt4 ) and 5 promoter regions were detected by bisulfite mutagenesis and sequencing as previously described (15). Loci locations are listed below: *LINE-1* (875-1156bp, GeneBank acc.no. D84391; 10 CpG sites), *Wnt4* (18687–18900bp, GeneBank acc.no. NC_000070; 14 CpG sites), *Ahcy* (chr2: 155075400-155075850; 6 CpG sites), *Apanc5* (chr5:122822280-122822880; 4 CpG sites), *Phgdh* (chr3: 98340932:98341557; 11 CpG sites), *Spns1* (chr7:126379283-126379734; 5 CpG sites), *Xylb* (chr9:119356730-119356989; 4 CpG sites). We used nest PCR and set the cycling conditions as denaturation for 5 min at 95 °C, followed by 35 cycles of 30s at 95 °C, 45s at 50 °C, and 30s at 72°C. The PCR products purified from agarose gel after electrophoresis were ligated into the TA cloning vectors (TaKaRa, pMD19-T), and at least 15 clones for each sample were sequenced. The methylation level of each sample was calculated by online tool QUMA web server (http://quma.cdb.riken.jp/) as described (16). The related primer sequences are listed in Supplemental Table 1.

**RNA-sequencing and data analysis.** Mouse morula embryos (7 embryos per sample, 2 samples for each group) were lysed and subjected to first strand synthesis as described previously (17). RNA-Seq library was prepared according to the instruction of KAPA Hyper Prep Kit (KAPA Biosystems). Libraries were sequenced with paired-end 150-bp reads on an Illumina HiSeq 2500 platform. RNA-Seq data were mapped to mm10 reference genome using STAR with parameters‘--two pass Mode Basic --sjdbGTFfile refFlat_mm10.gtf--out SAM type BAM Unsorted’. HT-Seq count was used to generate gene count matrix of each sample with parameters ‘-f bam -r name -s no’. The transcriptome reference used in mapping and quantification process is mm10 refFlat gtf. Differential gene expression analysis was performed using DESeq2 with |log2Foldchange| >1 and adjusted p value < 0.05. GO enrichment analysis was conducted by using EnrichR, and results were filtered with FDR < 0.05.

**Metabolomic profiling**

Metabolomic profiling for oocytes was conducted as we described previously (18). In brief, MII oocytes were collected from WT and Polg^m^ mice (300 oocytes per sample, 2 samples for each group). Samples were transferred to Eppendorf tubes after sharp freeze in liquid nitrogen, and stored at -80°C. For metabolite extraction, each sample was resuspended in 300 μl methanol: water (4:1) and homogenized using Ultra-Turrax homogenizer. Supernatant was transferred to a new tube, and dried. The metabolomics data were collected using an UPLC Ultimate 3000 system (Dionex, Germering, Germany) coupled to a Q-Exactive mass spectrometer (Thermo Fisher Scientific, Bremen, Germany). The mass spectrometer was performed in a full- scan mode ranging from 70 m/z to 1050 m/z, running at a 70,000 resolution in both positive and negative modes simultaneously.

Raw data acquired by the mass spectrometer were submitted to TraceFinder (v3.1). The metabolite identification was conducted by the comparison of accurate mass and retention time with the standard compounds. All statistical analyses were performed using “R” (V2.15). Student’s t test was used to compare continuous variables between two groups (Kim, 2015). SIMCA-P software (V14.0; Umetrics AB, Umea, Sweden) was employed for OPLS-DA. The variable importance in projection (VIP) 865 value > 1.00 and P value < 0.2 with a 1.3-fold change of each metabolite were used as the combined cut-offs of the statistical significance. This combination criteria allow to find more biologically meaningful sets of metabolites than P value alone (19).

**Statistical analysis.** All experiments were repeated three times with similar results, and data from one representative experiment are shown unless otherwise stated. All analyses were performed using GraphPad Prism (version 7.0) for Windows. Statistical comparisons were made with Student’s *t* test unless otherwise indicated. Data are presented as mean value ± SD. Changes were considered statistically significant when *P* < 0.05.

1. Kujoth, GC, Hiona, A, Pugh, TD*, et al.* Mitochondrial DNA mutations, oxidative stress, and apoptosis in mammalian aging. *Science*. 2005; **309**(5733): 481-4.

2. Han, L, Ren, C, Li, L*, et al.* Embryonic defects induced by maternal obesity in mice derive from Stella insufficiency in oocytes. *Nat Genet*. 2018; **50**(3): 432-42.

3. Zhang, Z, He, C, Zhang, L*, et al.* Alpha-ketoglutarate affects murine embryo development through metabolic and epigenetic modulations. *Reproduction*. 2019; **158**(2): 123-33.

4. Santos, F, Hendrich, B, Reik, W*, et al.* Dynamic reprogramming of DNA methylation in the early mouse embryo. *Dev Biol*. 2002; **241**(1): 172-82.

5. Wang, Q, Ratchford, AM, Chi, MM*, et al.* Maternal diabetes causes mitochondrial dysfunction and meiotic defects in murine oocytes. *Mol Endocrinol*. 2009; **23**(10): 1603-12.

6. Hou, X, Zhang, L, Han, L*, et al.* Differing roles of pyruvate dehydrogenase kinases during mouse oocyte maturation. *J Cell Sci*. 2015; **128**(13): 2319-29.

7. Smallwood, SA, Lee, HJ, Angermueller, C*, et al.* Single-cell genome-wide bisulfite sequencing for assessing epigenetic heterogeneity. *Nature methods*. 2014; **11**(8): 817-20.

8. Krueger, F, Andrews, SR. Bismark: a flexible aligner and methylation caller for Bisulfite-Seq applications. *Bioinformatics*. 2011; **27**(11): 1571-2.

9. O'Leary, NA, Wright, MW, Brister, JR*, et al.* Reference sequence (RefSeq) database at NCBI: current status, taxonomic expansion, and functional annotation. *Nucleic Acids Res*. 2016; **44**(D1): D733-45.

10. Jurka, J. Repbase update: a database and an electronic journal of repetitive elements. *Trends Genet*. 2000; **16**(9): 418-20.

11. Jühling, F, Kretzmer, H, Bernhart, SH*, et al.* metilene: fast and sensitive calling of differentially methylated regions from bisulfite sequencing data. *Genome Res*. 2016; **26**(2): 256-62.

12. Dennis, G, Jr., Sherman, BT, Hosack, DA*, et al.* DAVID: Database for Annotation, Visualization, and Integrated Discovery. *Genome Biol*. 2003; **4**(5): P3.

13. Veselovska, L, Smallwood, SA, Saadeh, H*, et al.* Deep sequencing and de novo assembly of the mouse oocyte transcriptome define the contribution of transcription to the DNA methylation landscape. *Genome Biol*. 2015; **16**: 209.

14. Wang, L, Zhang, J, Duan, J*, et al.* Programming and inheritance of parental DNA methylomes in mammals. *Cell*. 2014; **157**(4): 979-91.

15. Borghol, N, Lornage, J, Blachere, T*, et al.* Epigenetic status of the H19 locus in human oocytes following in vitro maturation. *Genomics*. 2006; **87**(3): 417-26.

16. Kumaki, Y, Oda, M, Okano, M. QUMA: quantification tool for methylation analysis. *Nucleic Acids Res*. 2008; **36**(Web Server issue): W170-5.

17. Picelli, S, Bjorklund, AK, Faridani, OR*, et al.* Smart-seq2 for sensitive full-length transcriptome profiling in single cells. *Nat Methods*. 2013; **10**(11): 1096-8.

18. Li, L, Zhu, S, Shu, W*, et al.* Characterization of Metabolic Patterns in Mouse Oocytes during Meiotic Maturation. *Mol Cell*. 2020.

19. McCarthy, DJ, Smyth, GK. Testing significance relative to a fold-change threshold is a TREAT. *Bioinformatics*. 2009; **25**(6): 765-71.
